# Topography of mutational signatures in human cancer

**DOI:** 10.1016/j.celrep.2023.112930

**Published:** 2023-08-04

**Authors:** Burçak Otlu, Marcos Díaz-Gay, Ian Vermes, Erik N. Bergstrom, Maria Zhivagui, Mark Barnes, Ludmil B. Alexandrov

**Affiliations:** 1 Department of Cellular and Molecular Medicine, UC San Diego, La Jolla, CA 92093, USA; 2 Department of Bioengineering, UC San Diego, La Jolla, CA 92093, USA; 3 Moores Cancer Center, UC San Diego, La Jolla, CA 92037, USA; 4 Department of Health Informatics, Graduate School of Informatics, Middle East Technical University, Ankara 06800, Turkey; 5 COSMIC, Wellcome Sanger Institute, Hinxton, Cambridgeshire CB10 1SA, UK; 6 Lead contact

## Abstract

The somatic mutations found in a cancer genome are imprinted by different mutational processes. Each process exhibits a characteristic mutational signature, which can be affected by the genome architecture. However, the interplay between mutational signatures and topographical genomic features has not been extensively explored. Here, we integrate mutations from 5,120 whole-genome-sequenced tumors from 40 cancer types with 516 topographical features from ENCODE to evaluate the effect of nucleosome occupancy, histone modifications, CTCF binding, replication timing, and transcription/replication strand asymmetries on the cancer-specific accumulation of mutations from distinct mutagenic processes. Most mutational signatures are affected by topographical features, with signatures of related etiologies being similarly affected. Certain signatures exhibit periodic behaviors or cancer-type-specific enrichments/depletions near topographical features, revealing further information about the processes that imprinted them. Our findings, disseminated via the COSMIC (Catalog of Somatic Mutations in Cancer) signatures database, provide a comprehensive online resource for exploring the interactions between mutational signatures and topographical features across human cancer.

## In brief

Comprehensive topography analysis of mutational signatures encompassing 82,890,857 somatic mutations in 5,120 whole-genome-sequenced tumors across 40 cancer types. Otlu et al. provide an online resource, through the COSMIC signatures database, that allows researchers to explore the interactions between somatic mutational processes and genome architecture within and across cancer types.

## Graphical Abstract

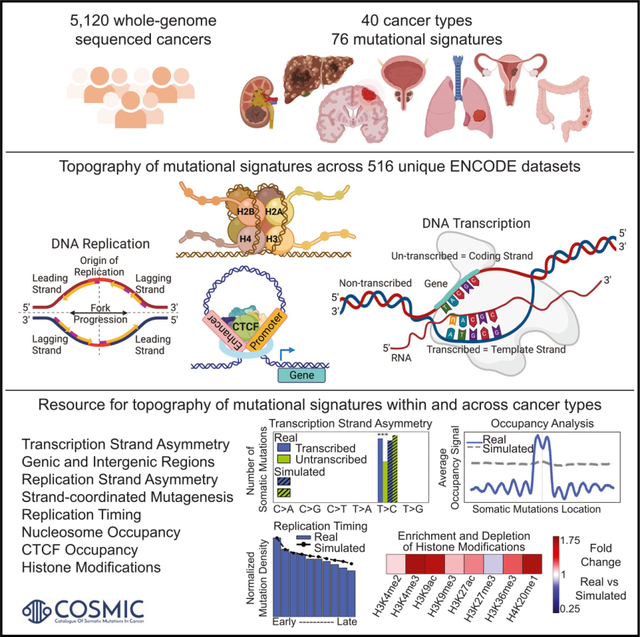

## INTRODUCTION

Cancer genomes are peppered with somatic mutations imprinted by the activities of different endogenous and exogenous mutational processes.^1,2^ Due to their intrinsic biophysical and biochemical properties, each mutational process engraves a characteristic pattern of somatic mutations, known as a mutational signature.^3^ Our previous analyses encompassing more than 5,000 whole-genome- and 20,000 whole-exome-sequenced human cancers have revealed the existence of at least 78 single-base substitution (SBS), 11 doublet-base substitution (DBS), and 18 insertion or deletion (ID) mutational signatures.^4–7^ Through statistical associations and further experimental characterizations, etiology has been proposed for approximately half of the identified signatures.^4,8–15^ Prior studies have also explored the interactions between somatic mutations imprinted by different mutational processes and the topographical features of the human genome for certain cancer types and for a small subset of topographical features. However, previously, there has been no comprehensive evaluation that examined the effect of genome architecture and topographical features on the accumulation of somatic mutations from different mutational signatures across human cancer.

Early studies have shown that late-replicating regions and condensed chromatin regions accumulate more mutations when compared with early-replicating regions, actively transcribed regions, and open chromatin regions.^16–19^ Subsequent analyses of hundreds of cancer genomes have revealed that differential DNA repair can explain variations in mutation rates across some cancer genomes^20^ as well as that chromatin features originating from the cell of origin, which gave rise to the tumor, can affect mutation rate and the distribution of somatic mutations.^17^ Recently, Morganella et al. examined the effect of the genomic and the epigenomic architecture on the activity of 12 SBS signatures in breast cancer.^21^ These analyses demonstrated that mutations generated by different mutational processes exhibit distinct strand asymmetries and that mutational signatures are differently affected by replication timing and nucleosome occupancy.^21^ Pan-cancer exploration of strand asymmetries was also conducted for different mutation types across multiple cancer types,^22^ as well as for different mutational signatures.^23^ In particular, pan-cancer analyses of more than 3,000 cancers have revealed the strand asymmetries and replication timings of the 30 SBS mutational signatures from the Catalog of Somatic Mutations in Cancer v.2 signatures database (COSMICv.2).^23^ Similarly, more than 3,000 cancer genomes were used to elucidate the effect of nucleosome occupancy for the 30 substitution signatures from COSMICv.2.^24^ More recently, a study has also shown the interplay between the three-dimensional genome organization and the activity of certain mutational signatures.^25,26^

Here, we report the most comprehensive evaluation of the effect of nucleosome occupancy, histone modifications, CCCTC-binding factor (CTCF) binding sites, replication timing, transcription strand asymmetry, and replication strand asymmetry on the cancer-specific accumulation of somatic mutations from distinct mutational signatures. Our analysis leverages the complete set of known COSMICv.3.3 signatures (78 SBS, 11 DBS, and 18 ID), and it examines 5,120 whole-genome-sequenced cancers while simultaneously utilizing 516 unique tissue-matched topographical features from the ENCODE project (Table S1).^27^ In all analyses, the observed patterns of somatic mutations are compared to background simulation models of mutational signatures that mimic both the trinucleotide pattern of these signatures as well as their mutational burden within each chromosome in each examined sample (STAR Methods). Our results confirm many of the observations previously reported for strand asymmetry, replication timing, and nucleosome periodicity for the original COSMICv.2 signatures. Further, the richer and larger COSMICv.3.3 dataset allowed us to elucidate novel biological findings for some of these 30 SBS signatures, revealing previously unobserved pan-cancer and cancer-specific dependencies. Additionally, this resource provides the first-ever map of the genome topography of ID, doublet-base, and another 24 substitution signatures in human cancer. Moreover, our study is the first to examine the tissue-specific effect of CTCF binding and 11 different histone modifications on the accumulation of somatic mutations from different mutational signatures. As part of the results, we provide a global view of the topography of mutational signatures across 5,120 whole-genome-sequenced tumors from 40 types of human cancer, and we include cancer-type-specific examples. As part of the discussion, we zoom in on two distinct case studies: (1) the topography of different types of clustered somatic mutations and (2) using the topography of mutational signatures to separate mutational signatures with similar patterns. Lastly, the reported results are released as part of the COSMICv.3.3 signatures database, https://cancer.sanger.ac.uk/signatures, providing an unprecedented online resource for examining the topography of mutational signatures within and across human cancer types.

## RESULTS

### Transcription strand asymmetries

Transcription strand asymmetries have been generally attributed to transcription-coupled nucleotide excision repair (TC-NER) since bulky adducts (e.g., ones due to tobacco carcinogens) in actively transcribed regions of the genome will be preferentially repaired by TC-NER.^28^ Additionally, TC damage may also lead to transcription strand asymmetry due to one of the strands being preferentially damaged during transcription.^22^

**Figure 1.**
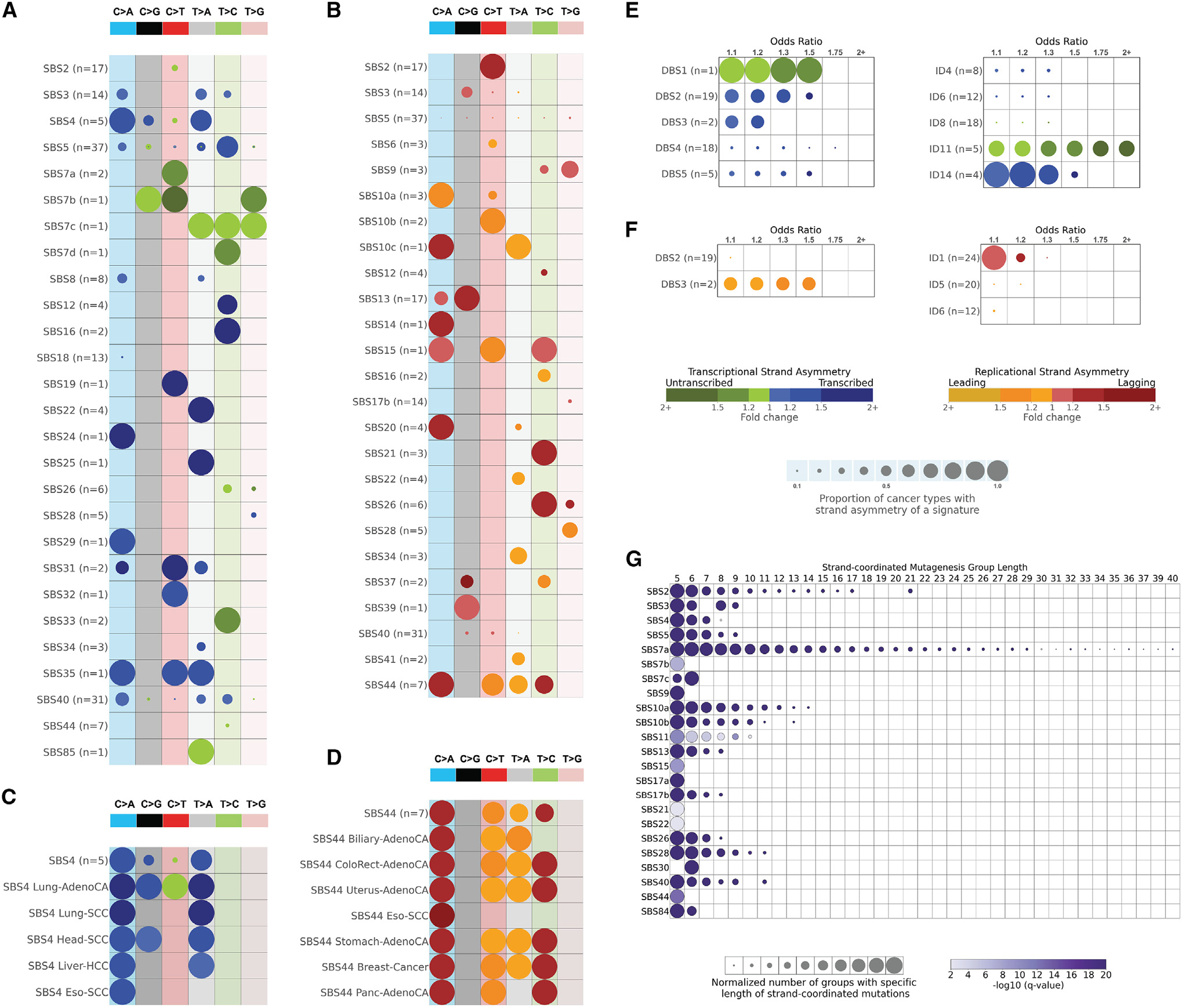
Strand asymmetries and strand-coordinated mutagenesis (A) Transcription strand asymmetries of signatures of single-base substitutions (SBSs). Rows represent the signatures, where *n* reflects the number of cancer types in which each signature was found. Columns display the six substitution subtypes based on the mutated pyrimidine base: C>A, C>G, C>T, T>A, T>C, and T>G. SBS signatures with transcription strand asymmetries on the transcribed and/or the untranscribed strands with adjusted p values ≤ 0.05 (Fisher’s exact test corrected for multiple testing using Benjamini-Hochberg) are shown in circles with blue and green colors, respectively. The color intensity reflects the odds ratio between the ratio of real mutations and the ratio of simulated mutations, where each ratio is calculated using the number of mutations on the transcribed strand and the number of mutations on the untranscribed strand. Only odds ratios above 1.10 are shown. Circle sizes reflect the proportion of cancer types exhibiting a signature with specific transcription strand asymmetry. (B) Replication strand asymmetries of SBS signatures. Rows represent the signatures, where *n* reflects the number of cancer types in which each signature was found. Columns display the six substitution subtypes based on the mutated pyrimidine base: C>A, C>G, C>T, T>A, T>C, and T>G. SBS signatures with replicational strand asymmetries on the lagging strand or on the leading strand with adjusted p values ≤ 0.05 (Fisher’s exact test corrected for multiple testing using Benjamini-Hochberg) are shown in circles with red and yellow colors, respectively. The color intensity reflects the odds ratio between the ratio of real mutations and the ratio of simulated mutations, where each ratio is calculated using the number of mutations on the lagging strand and the number of mutations on the leading strand. Only odds ratios above 1.10 are shown. Circle sizes reflect the proportion of cancer types exhibiting a signature with specific replication strand asymmetry. (C) Transcription strand asymmetries of signature SBS4 across cancer types. Data are presented in a format similar to the one in (A). (D) Replication strand asymmetries of signature SBS44 across cancer types. Data are presented in a format similar to the one in (B). (E) Transcription strand asymmetries of signatures of doublet-base substitutions (DBSs) and of small insertions or deletions (IDs). Data are presented in a format similar to the one in (A). (F) Replication strand asymmetries of DBS and ID mutational signatures. Data are presented in a format similar to the one in (B). (G) Strand-coordinated mutagenesis of SBS signatures. Rows represent SBS signatures and columns reflect the lengths, in numbers of consecutive mutations, of strand-coordinated mutagenesis groups. SBS signatures with statistically significant strand-coordinated mutagenesis (adjusted p values ≤ 0.05, z-test corrected for multiple testing using Benjamini-Hochberg) are shown as circles under the respective group length with a minimum length of 5 consecutive mutations. The size of each circle reflects the number of consecutive mutation groups for the specified group length normalized for each signature. The color of each circle reflects the statistical significance of the number of subsequent mutation groups for each group length with respect to simulated mutations. See also Figure S1.

Mutational signatures with similar etiologies generally exhibited consistent patterns of transcription strand asymmetries across cancer types. Specifically, most signatures attributed to exogenous mutational processes showed transcription strand bias with mutations usually enriched on the transcribed strand (Figures 1A and 1E). This included signatures SBS4/DBS2 (both previously attributed to mutagens in tobacco smoke), SBS16 (alcohol consumption), SBS24 (aflatoxin), SBS29 (tobacco chewing), SBS25/SBS31/SBS35/DBS5 (prior chemotherapy), and SBS32 (prior treatment with azathioprine). Nevertheless, for some exogenous signatures, strand asymmetries could differ between cancer types. For example, while transcriptional asymmetries for C>A and T>A mutations from SBS4 were observed across most cancer types, asymmetries for C>G mutations were only observed in lung adenocarcinoma and cancers of the head and neck (Figure 1C). Interestingly, C>T mutations attributed to SBS4 had strand asymmetry only in lung adenocarcinoma. In contrast, mutational signatures due to direct damage from ultraviolet light (viz., SBS7a/b/c/d and DBS1) were the only known exogenous mutational processes to exhibit transcription strand asymmetry with strong enrichment of mutations on the untranscribed strand, consistent with damage from ultraviolet light on cytosine (Figures 1A and 1E).

Transcription strand asymmetry with consistent enrichment of mutations on the transcribed strand was also observed for clock-like signature SBS5 and for multiple mutational signatures with unknown etiology, including SBS12, SBS19, and ID14 (Figures 1A and 1E). Strand bias with preferences for the untranscribed strand was observed for signatures ID11 and SBS33 (both with unknown etiology). Lastly, other mutational signatures exhibited transcription strand asymmetry in only a small subset of cancer types (Figures 1A and 1E).

### Mutational signatures in genic and intergenic regions

Except for SBS16 and ID11, all other signatures were enriched in intergenic regions across most cancer types, with the enrichment ranging from 1.30-fold (e.g., SBS24) to more than 2-fold (e.g., SBS17a/b; Figures S1A–S1C). The observed depletion of mutations in genic regions was not due to transcription strand asymmetries, as correcting the asymmetries, by assigning the number of mutations on both transcribed and untranscribed strands to their highest value, resulted in only minor alterations of the fold change increases (Figures S1D and S1E). Overall, these results suggest that transcription strand asymmetry, usually attributed to the activity of TC-NER, does not account for the high enrichment of somatic mutations in intergenic regions.

SBS16 and ID11 showed enrichment of mutation in genic regions in liver and esophageal cancers, while ID11 was also enriched in genic regions in cancers of the head and neck. SBS16 has been previously associated with exposure to alcohol^29–31^ and has been attributed to the activity of TC damage.^22^ Prior studies have also associated ID11 with alcohol consumption in esophageal cancers.^7^ Re-examining ID11 in the current cohort of whole-genome-sequenced liver cancers, by comparing the mutations attributed to ID11 in 32 heavy drinkers with the mutations attributed to ID11 in 94 light drinkers, reveals a 2-fold enrichment in heavy drinkers (p = 1.31 × 10^−3^; Mann-Whitney Utest). This and the prior associations in esophageal cancers^7^ strongly suggest a similar exogenous mutational processes, related to alcohol consumption, accounting for the enrichment of mutation in genic regions for both signatures SBS16 and ID11.

### Replication strand asymmetries

Replication strand bias was consistently observed in most signatures attributed to aberrant or defective endogenous mutational processes with strand bias either on the leading or on the lagging strand (Figures 1B and 1F). Strong replication strand asymmetries with enrichment of mutations on the leading strand were observed for signatures previously attributed to the defective activity of polymerases, including (1) SBS10a/SBS10b/DBS3 found in samples with exonuclease domain mutations in DNA polymerase epsilon (*POLE*); (2) SBS9, attributed to infidelity of polymerase eta (POLH); and (3) SBS10c due to defective polymerase delta (*POLD1*). Interestingly, SBS28 (unknown etiology) exhibited a strong replication strand bias when found at high levels in POLE-deficient samples.

Mutational signatures associated with defective DNA mismatch repair exhibited statistically significant replication strand bias either predominately on the leading strand (viz., SBS6) or on the lagging strand (viz., SBS14, SBS15, SBS20, SBS21, SBS26, SBS44, ID1). There were some minor inconsistencies of replication strand bias across cancer types. For example, SBS44 did not have replication strand asymmetry for C>T, T>A, and T>C mutations in esophageal squamous cell carcinoma (Figure 1D). Somatic mutations due to signatures SBS2 and SBS13, both attributed to the aberrant behavior of the APOBEC3 family of deaminases,^32^ were found enriched on the lagging strand in all cancer types. This result is consistent with the observation that single-stranded DNA formed during DNA replication on the lagging strand is a major substrate for the APOBEC3 family of deaminases.^33,34^ Lastly, several other mutational signatures, most with unknown etiology, exhibited replication strand bias within a small set of cancer types (Figures 1B and 1F).

### Strand-coordinated mutagenesis

Prior analyses have shown that certain types of mutations on the same reference allele were observed on the same strand more frequently than expected by chance.^21,34,35^ These strand-coordinated clustered mutations usually arise due to damage on single-stranded DNA, and they are often indicative of the formation of hypermutable loci in the genome.^33,34^

SBS7a, attributed to ultraviolet (UV) light, attained the highest strand-coordinated mutagenesis with lengths of subsequent mutations up to 40 consecutive mutations (Figure 1G). In contrast, other mutational signatures attributed to UV light, mainly SBS7b/c/d, either did not exhibit or exhibited much lower strand-coordinated mutagenesis. APOBEC3-attributed SBS2 and SBS13 showed strand-coordinated mutagenesis with as many as 21 consecutive strand-coordinated mutations. Additionally, strand-coordinated mutations were observed for SBS17b (unknown etiology), SBS10a/b (*POLE* deficiency), SBS4 (tobacco smoking), SBS26 (defective mismatch repair), and SBS28 (unknown etiology).

### The effect of DNA replication timing

**Figure 2.**
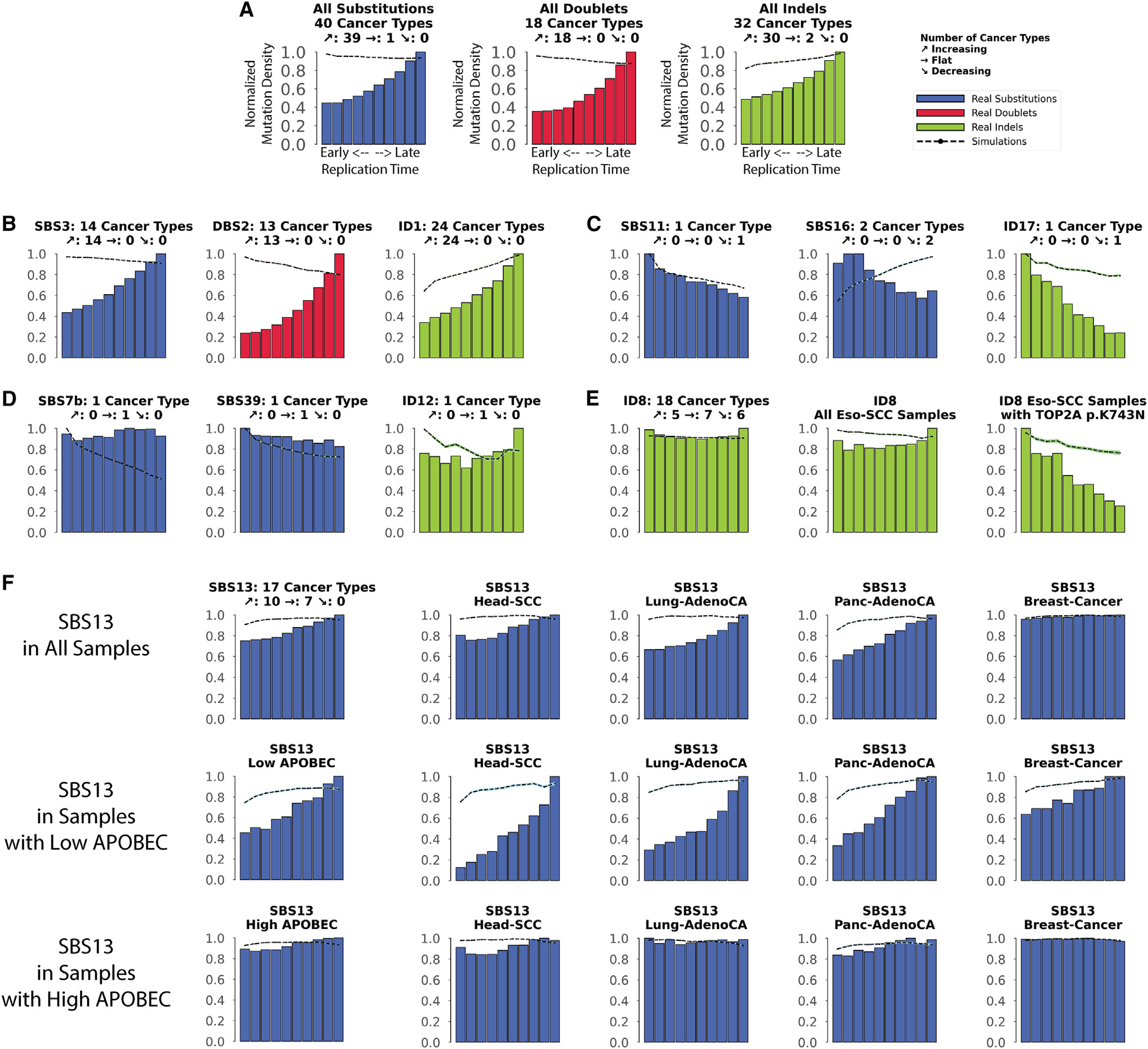
Interplay between replication timing and mutational signatures Replication time data are separated into deciles, with each segment containing exactly 10% of the observed replication time signal (x axes). Normalized mutation densities per decile (y axes) are presented for early (left) to late (right) replication domains. Real data for SBS signatures are shown as blue bars, for DBS signatures as red bars, and for small ID signatures as green bars. Simulated somatic mutations are shown as dashed lines. Where applicable, the total number of evaluated cancer types for a particular mutational signature is shown on top of each plot (e.g., 18 cancer types were evaluated for ID8 in E). For each signature, the number of cancer types where the mutation density increases with replication timing is shown next to the slanted up arrow (↗; e.g., 5 cancer types for ID8). Similarly, the number of cancer types where the mutation density decreases with replication timing is shown next to the slanted down arrow (↘; e.g., 6 cancer types for ID8). Lastly, the number of cancer types where the mutation density is not affected by replication timing is shown next to the right-pointing arrow (→ e.g., 7 cancer types for ID8). (A) All SBSs, DBSs, and IDs across all examined cancer types with each cancer type examined separately. (B) Exemplar signatures consistently associated with late replication timing. (C) Exemplar signatures consistently associated with early replication timing. (D) Exemplar signatures consistently unaffected by replication timing. (E) ID8 as a mutational signature inconsistently affected by replication timing. (F) The effect of replication timing on APOBEC3-associated signature SBS13 in samples with low and high APOBEC3 mutational burden. See also Figure S2.

Consistent with prior reports,^18,36–38^ the aggregated set of somatic mutations was shown to be enriched in late-replicating regions for most cancer types (Figure 2A). Specifically, from the examined 40 cancer types, SBSs were found to be more common in regions of the genome that undergo late replication in 39/40 cancer types and were not associated with replication only in uveal melanoma (Figure 2A). Similarly, DBSs and IDs were enriched in late-replicating regions in 18/18 and 30/32 cancer types, respectively. Note that due to their lower mutational burdens, we could confidently evaluate DBSs and IDs only in a subset of cancer types. In agreement with the aggregated analysis, most mutational signatures imprinted somatic mutations with an increased normalized mutational density from early- to late-replicating regions (Figure S2). For example, SBS3 (defective homologous recombination) was enriched in late-replicating regions in all 14 cancer types where the signature can be confidently evaluated. Other examples include signatures DBS2 and ID1, which were also consistently enriched in all examined cancer types (Figure 2B).

Nevertheless, at least seven mutational signatures were found predominately enriched in early-replicating regions, including ID17, likely due to *TOP2A* mutations; SBS11, due to temozolomide therapy; SBS16 and ID11, both associated with alcohol consumption; SBS6 and SBS15, both attributed to mismatch repair deficiency; and SBS84 due to the aberrant activities of activation-induced (AID) cytidine deaminases (Figures 2C and S2). Moreover, multiple mutational signatures were generally unaffected by replication timing, including SBS7b (UV light); SBS20, SBS21, and SBS44 (attributed to failure of mismatch repair); SBS30 (deficient base excision repair); and SBS39 and ID12 (unknown etiology; Figures 2D and S2). The lack of association with replication timing for some of these mutational signatures can be potentially attributed to the activity of DNA translesion polymerases.^39,40^

Interestingly, a number of mutational signatures exhibited cancer-type-specific associations with replication timing (Figures 2E and Figure S2). For example, signature ID8 was enriched with replication timing in 5 cancer types, was depleted in 6 cancer types, and was unaffected by replication timing in 7 cancer types (Figure 2E). Multiple etiologies have been associated with ID8,^4,41^ including mutations resulting in K743N amino acid change in TOP2A. All samples harboring such mutations in *TOP2A* exhibited an enrichment of ID8 in early-replicating regions (Figure 2E). The other cancer-type-specific mechanisms resulting in different associations with replication timing for ID8 remain unknown.

Another notable example of cancer-type-specific associations with replication timing is the APOBEC3-associated SBS13 (Figure S2). SBS13 showed no dependence on replication timing in 7/17 cancer types (viz., bladder, breast, uterus, cervix, ovary, thyroid, and acute lymphocytic leukemia; Figure 2F). This behavior is consistent with prior reports where SBS13 was attributed to uracil excision of deaminated cytosine followed by processing by DNA translesion polymerases in breast cancer.^39,40^ Surprisingly, in 10/17 cancer types, SBS13 was highly enriched in late-replicating regions. Using a previously defined approach for separating the cancer samples into ones where SBS13 is not a hypermutator (low APOBEC3) and ones where SBS13 is a hypermutator (high APOBEC3) revealed that the lack of dependence on replication timing is predominately characteristic for hypermutated samples (Figure 2F). This result indicates that DNA translesion polymerases may play a significantly larger role in APOBEC3 hypermutators than previously anticipated.

### The effect of nucleosome occupancy

Nucleosomes are the basic packing units of chromatin, with each nucleosome consisting of ~147 base pair (bp) DNA wrapped around a histone octamer with 60–80 bp linker DNA between consecutive nucleosomes.^42,43^ Previous analyses have revealed dependencies between mutational signatures operative in breast cancer and nucleosome occupancy^21^ as well as a pan-cancer periodicity of mutation rates within nucleosomes due to multiple substitution signatures.^24^ However, beyond breast cancer, there has been no cancer-specific examination of the effect of nucleosome occupancy on different mutational signatures.

**Figure 3.**
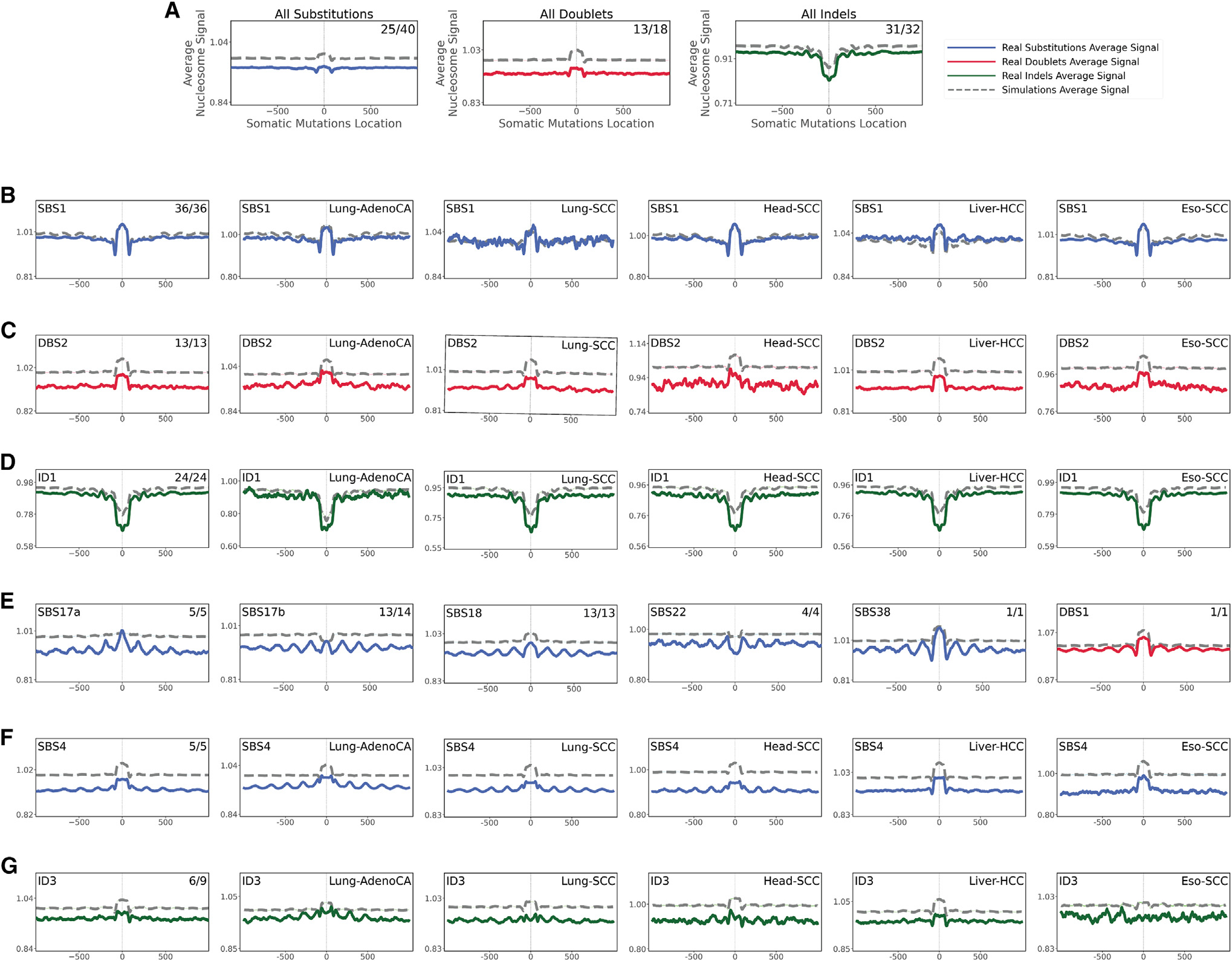
Relationship between mutational signatures and nucleosome occupancy In all cases, solid lines correspond to real somatic mutations, with blue solid lines reflecting SBSs, red solid lines reflecting DBSs, and green solid lines reflecting small IDs. Simulated somatic mutations are shown as dashed lines. Solid lines and dashed lines display the average nucleosome signal (y axes) along a 2 kb window (x axes) centered at the mutation start site for real and simulated mutations, respectively. The mutation site is annotated in the middle of each plot and denoted as 0. The 2 kb window encompasses 1,000 base pairs 5′ adjacent to each mutation as well as 1,000 base pairs 3′ adjacent to each mutation. Where applicable, the total number of similar and considered cancer types using an *X/Y* format, with *X* being the number of cancer types where a signature has similar nucleosome behavior (Pearson correlation ≥ 0.5 and adjusted p value ≤ 0.05, z-test corrected for multiple testing using Benjamini-Hochberg) and Y representing the total number of examined cancer types for that signature. For example, signature ID3 in (G) annotated with 6/9 reflects a total of 9 examined cancer types with similar nucleosome behavior observed in 6 cancer types. (A) All SBSs, DBSs, and IDs across all examined cancer types with each cancer type examined separately. (B–D) The nucleosome occupancy of signatures SBS1 (B), DBS2 (C), and ID1 (D) are shown across all cancer types as well as within cancers of the lung, head and neck, liver, and esophagus. (E) Signatures with consistent periodicities of mutation rates around the nucleosome. (F and G) Tobacco-associated SBS4 (F) and ID3 (G) exhibiting periodicities of mutation rates only in certain cancer types. See also Figure S3.

Aggregated somatic mutations and mutations attributed to most mutational signatures were depleted near nucleosomes compared to simulated data that mimic the mutational landscapes of the examined cancer genomes (Figure 3A). Remarkably, the majority of SBS, DBS, and ID mutational signatures were similarly affected by nucleosome occupancy across most cancer types (Figure S3). Some signatures were consistently enriched in the vicinity of nucleosomes. For example, clock-like signature SBS1 exhibited a pattern closely mimicking simulated data and showing a higher number of mutations at nucleosomes in 36/36 cancer types, including cancers of the lung, head and neck, liver, and esophagus (Figure 3B). In contrast, some signatures were markedly different from the simulated data (Figure S3), including signature DBS2, which was consistently depleted across 13/13 cancer types (Figure 3C). Moreover, some signatures were depleted in nucleosomes and, strikingly, appeared at linker DNA (Figure S3). For example, clock-like signature ID1 was depleted when compared to simulated data, and it exhibited depletion in nucleosomes in 24/24 of the examined cancer types (Figure 3D). The mutations engraved by most flat mutational signatures (e.g., SBS5, SBS8, SBS40) were generally unaffected by nucleosomes (Figure S3).

Different types of periodicities of mutation rates around the nucleosome structure were observed for signatures associated with tobacco smoking (SBS4 and ID3), UV light (SBS7a/b/c/d), POLE deficiency (SBS10a), aristolochic acid (SBS22), and reactive oxygen species (SBS18, SBS36, and SBS38; Figures 3E and S3). Interestingly, signatures SBS17a/b also showed similar periodic dependencies (Figure 3E), providing further circumstantial evidence for the hypothesis that SBS17a/b may also be due to reactive oxygen species damage of the deoxyribonucleoside triphosphate pools.^23,44–48^ With the exception of signatures SBS22 and ID3, all other periodic signatures exhibited enrichment of mutations at nucleosomes (Figures 3E and 3G). Further, for most signatures, periodicity of mutation rates was observed in each cancer type where the signature was operative (Figure S3). Nevertheless, signature SBS4 showed strong periodicity in cancers of the lung and head and neck but not in cancers of the liver or esophagus (Figure 3F). Similarly, signature ID3 exhibited periodic behavior only in cancers of the lung but not in any other cancer type (Figure 3G).

### The effect of CTCF binding

CTCF is a multi-purpose, sequence-specific DNA-binding protein with an essential role in transcriptional regulation, somatic recombination, and chromatin architecture.^49^ The human genome harbors many CTCF binding sites with prior studies reporting that mutations due to UV light are enriched in CTCF binding sites.^50^

**Figure 4.**
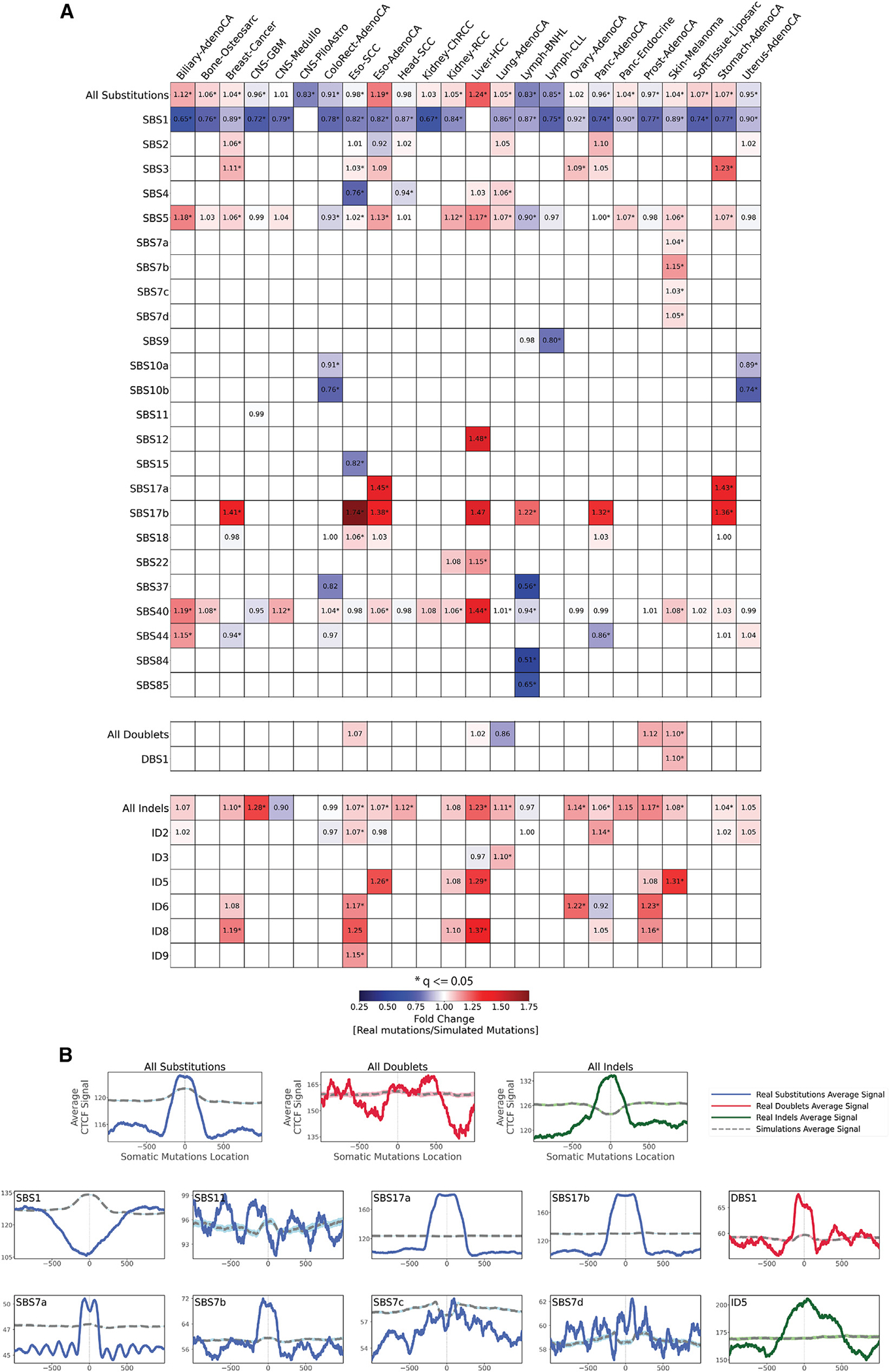
Relationship between mutational signatures and CTCF binding sites (A) Enrichments and depletions of somatic mutations within CTCF binding sites. Heatmaps display only mutational signatures and cancer types that have at least one statistically significant enrichment or depletion of somatic mutations attributed to signatures of either SBSs, DBSs, or small IDs. Red colors correspond to enrichments of real somatic mutations when compared to simulated data. Blue colors correspond to depletions of real somatic mutations when compared to simulated data. The intensities of red and blue colors reflect the degree of enrichments or depletions based on the fold change. White colors correspond to lack of data for performing statistical comparisons (e.g., signature not being detected in a cancer type). Statistically significant enrichments and depletions are annotated with an asterisk (*; adjusted p value ≤ 0.05, z-test combined with Fisher’s method and corrected for multiple testing using Benjamini-Hochberg). (B) The top three panels reflect average CTCF occupancy signal for all SBSs, DBS, and IDs across all examined cancer types. Bottom panels reflect all somatic mutations attributed for several exemplar mutational signatures across all cancer types. In all cases, solid lines correspond to real somatic mutations, with blue solid lines reflecting SBSs, red solid lines reflecting DBSs, and green solid lines reflecting IDs. Solid lines and dashed lines display the average CTCF binding signal (y axes) along a 2 kb window (x axes) centered at the mutation start site for real and simulated mutations, respectively. The mutation start site is annotated in the middle of each plot and denoted as 0. The 2 kb window encompasses 1,000 base pairs 5′ adjacent to each mutation as well as 1,000 base pairs 3′ adjacent to each mutation.

Somatic mutations exhibited clear patterns of both enrichment and/or periodicity for multiple mutational signatures and CTCF binding sites (Figure 4). While some signatures were consistently depleted at CTCF biding sites across the majority of cancer types when compared to simulated data (SBS1, SBS9, SBS10a/b, SBS15, SBS37, SBS84, and SBS85), others were commonly enriched (SBS3, SBS5, SBS7a/b/d, SBS12, SBS17a/b, SBS18, SBS22, and SBS40; DBS1; ID5, ID6, ID8, and ID9; Figure 4A).

Aggregated SBSs exhibited an inconsistent behavior across cancer types with enrichment in some cancers (e.g., liver cancers) and depletions in others (e.g., lymphomas). In contrast, IDs were enriched at CTCF binding sites in the majority of cancer types (Figure 4A). Remarkably, the effect of CTCF occupancy tended to be also consistent for many signatures with similar etiologies. Strong periodicities of mutation rates around CTCF binding sites were observed for UV-associated signature SBS7a but not for UV-associated signatures DBS1 and SBS7b/c/d (Figure 4B).

Mutations due to SBS9, associated with defective POLH-driven replication errors, and signatures SBS10a/b, found in samples with mutations in *POLE* and/or *POLD1*, were strikingly depleted at CTCF binding sites. Signature SBS15, associated with microsatellite instability, was strongly depleted at CTCF binding sites (Figure 4A).

Only one of the clock-like signatures, SBS1, exhibited a depletion of mutations at CTCF binding sites (Figure 4A), while simulated data indicated that SBS1 should be enriched at these sites (Figure 4B). Signature SBS3, attributed to defective homologous recombination, was highly elevated in CTCF binding sites for breast, ovarian, stomach, and esophageal cancers. Signatures SBS17a/b exhibited a striking enrichment at CTCF binding sites in all cancer types with a sufficient number of mutations from each signature (Figure 4A). SBS17a showed enrichment in stomach and esophageal cancers, while SBS17b showed enrichment for stomach, esophageal, breast, pancreatic cancers, and non-Hodgkin’s lymphomas. In contrast, simulated data indicate that CTCF binding should have no effect on the accumulation of mutations from signatures SBS17a/b (Figure 4B).

### The effect of histone modifications

Each nucleosome consists of four pairs of core histones: H2A, H2B, H3, and H4. Post-translational modifications of histone tails play a key role in regulating DNA replication, gene transcription, and DNA damage response.^51^ To evaluate the effect of histone modifications on the accumulation of mutations from different mutational signatures, we mapped the depletion or the enrichment of mutations compared to simulated data in the context of the tissue-specific positions of 11 histone modifications: (1) H2AFZ, a replication-independent member of the histone H2A family that renders chromatin accessible at enhancers and promoters, regulating transcriptional activation and repression^52^; (2) H3K4me1, a histone mark often associated with enhancer activity^53^; (3) H3K4me2, a histone post-translational modification enriched in *cis*-regulatory regions, including both enhancers and promoters^54^; (4) H3K4me3, a post-translational modification enriched in active promoters near transcription start sites^55^; (5) H3K9ac, associated with active gene promoters and active transcription^56^; (6) H3K9me3, a silencer and a typical mark of constitutive heterochromatin^57^; (7) H3K27ac, a histone modification generally contained at nucleosomes flanking enhancers^55^; (8) H3K27me3, which is repressive and associated with silent genes^58^; (9) H3K36me3, associated with transcribed regions and playing a role in regulating DNA damage repair^59^; (10) H3K79me2, detected in the transcribed regions of active genes^60^; and (11) H4K20me1, found in gene promoters and associated with gene transcriptional elongation and transcription activation.^61^

Aggregated substitutions, dinucleotides, and IDs exhibited dissimilar behavior for different histone modifications across cancer types (Figure S4). Aggregated substitutions were predominately depleted around H2AFZ, H3K4me2, H3K4me3, and H3K27ac in approximately half of the examined cancer types (Figure S4A). Aggregated doublets and IDs did not have any clear pan-cancer preference but showed cancer-type-specific enrichments and depletions (Figures S4B and S4C). In contrast, the majority of mutational signatures had generally similar behavior in the vicinity of different histone modifications, revealing that histone modifications have similar effects on mutagenesis across cancer types (Figure S4). Most SBS mutational signatures were either unaffected or were depleted near histone marks (Figure S4A). Notable exceptions were APOBEC3-associated signatures SBS2 and SBS13, AID-associated signatures SBS84 and SBS85, and *POLH*-attributed SBS9, which were generally enriched near most histone modifications (Figure S4A). Doublet signatures DBS1, DBS2, DBS3, DBS4, and DBS5 were also predominately depleted near most histone marks (Figure S4B). In contrast, signatures DBS7, DBS9, and DBS11 were highly enriched near most histone marks. Most ID mutational signatures were either unaffected or very highly enriched near histone marks (Figure S4C), with the only exceptions being depletions of (1) ID1 and ID6 near H2AZ, (2) ID3 in the vicinity of H3K4me3, (3) ID5 near H3K27me3, and (4) ID14 in the vicinity of H3K36me3. While enrichments and depletions of somatic mutations in the vicinity of histone marks were commonly observed for different mutational signatures (Figures S4A–S4C), there was no specific pattern of mutations within 1,000 bp for any of the examined histone modifications. Exemplars of typically observed patterns of enrichments, depletions, or no changes around different histone modifications are provided for signatures SBS7a and ID1 across several histone modifications (Figure S5D).

**Figure 5.**
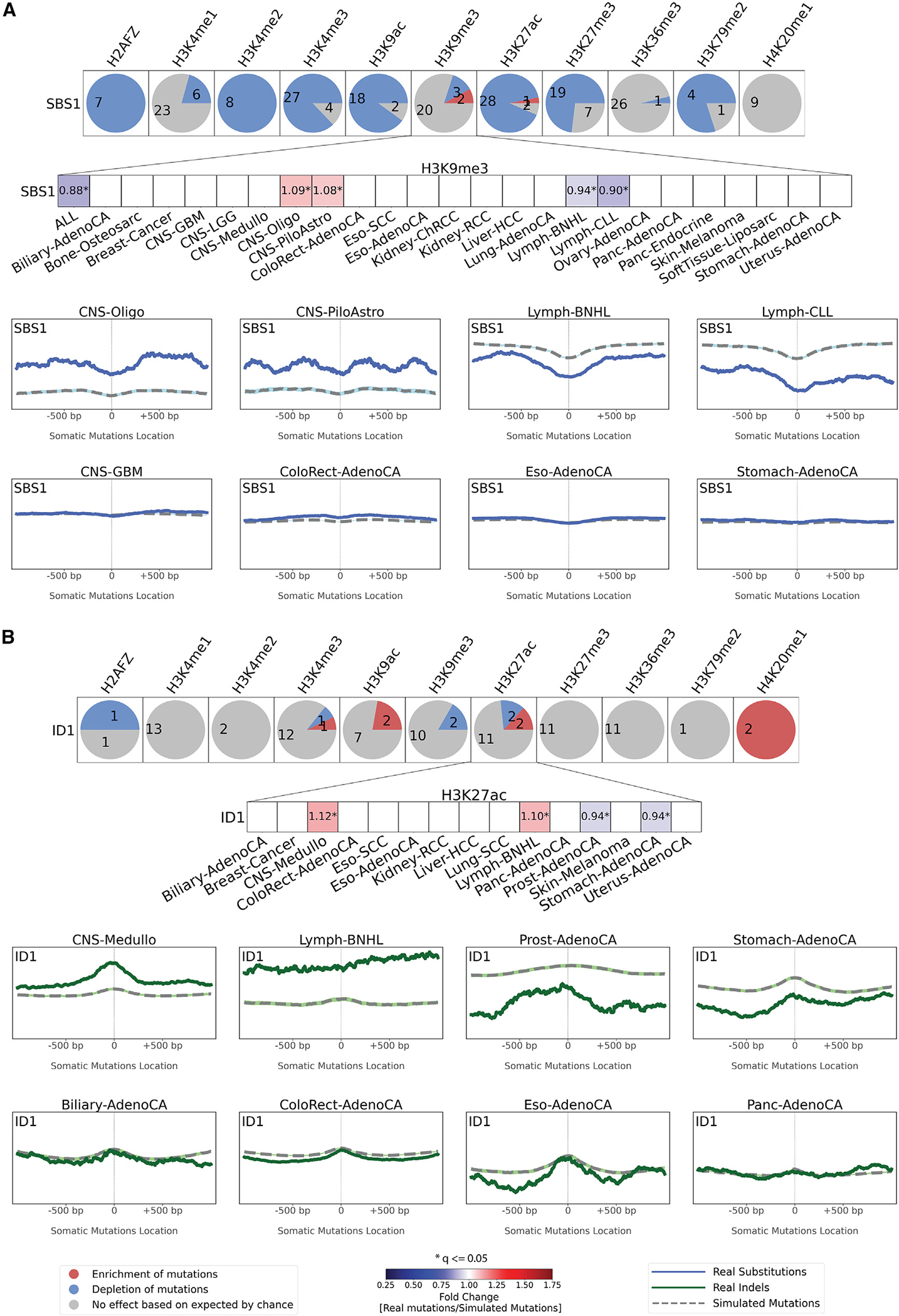
Exemplar relationships between mutational signatures and histone modifications The effect of histone modifications is shown for signatures SBS1 (A) and ID1 (B). For each signature, an evaluation was made for each of the 11 histone marks across all cancer types with sufficient numbers of somatic mutations with results presented as circles. Each circle is separated in red, blue, and gray segments proportional to the cancer types in which the signature has a specific behavior. A red segment in a circle reflects the signature being enriched in the vicinity of a histone modification (adjusted p value ≤ 0.05, z-test combined with Fisher’s method and corrected for multiple testing using Benjamini-Hochberg and at least 5% enrichment). A blue segment in a circle reflects the signature being depleted in the vicinity of a histone modification (adjusted p value ≤ 0.05, z-test combined with Fisher’s method and corrected for multiple testing using Benjamini-Hochberg and at least 5% depletion). A gray segment in a circle corresponds to neither depletion nor enrichment of the signature in the vicinity of a histone modification. The figure zooms in on the effect of H3K9me3 on SBS1 (A) and of H3K27ac on ID1 (B). Red colors correspond to enrichments of real somatic mutations when compared to simulated data. Blue colors correspond to depletions of real somatic mutations when compared to simulated data. The intensities of red and blue colors reflect the degree of enrichments or depletions based on the fold change. We further zoom in on the patterns of mutations around H3K9me3 and H3K27ac. Solid lines correspond to real somatic mutations, with blue solid lines reflecting SBSs and green solid lines reflecting IDs. Solid lines and dashed lines display the average histone mark signal (y axes) along a 2 kb window (x axes) centered at the mutation start site for real and simulated mutations, respectively. The mutation start site is annotated in the middle of each plot and denoted as 0. The 2 kb window encompasses 1,000 base pairs 5′ adjacent to each mutation as well as 1,000 base pairs 3′ adjacent to each mutation. See also Figure S4.

Next, we examine two mutational signatures that exhibited inconsistent enrichments and depletions near specific histone marks. Clock-like signature SBS1 was consistently depleted across cancer types for multiple histone marks, including H3K9me3 (Figure 5A). Nevertheless, SBS1 exhibited enrichment of mutations near H3K9me3 in two cancer types of the central nervous system, depletion of mutations near H3K9me3 in three hematological malignancies, and no effect in all other solid tumor types (Figure 5A). Similarly, signature ID1 exhibited dissimilar behavior near H3K27ac with enrichments in medulloblastoma and lymphoma, depletions in stomach and prostate cancer, and no change in most other cancer types (Figure 5B).

## DISCUSSION

Our analysis provides a comprehensive resource that maps the effects of topographical genomic features on the accumulation of somatic mutations from distinct mutational signatures. The reported results confirmed many of the prior observations for strand asymmetry, replication timing, and nucleosome periodicity for some of the original 30 COSMICv.2 SBS signatures.^21,23,24^ The examined larger dataset provided us with a greater resolution to identify previously unobserved pan-cancer and cancer-specific dependencies for some of these 30 signatures as well as to reveal the effect of genome architecture on the accumulation of another 46 mutational signatures across human cancer. Importantly, this report also provides the first-ever examination of the tissue-specific effect of CTCF binding and 11 different histone modifications on the accumulation of somatic mutations from different mutational signatures. In addition to the comprehensive global view in the results section, in this discussion, we zoom in on two specific case studies to further illustrate the power of using this resource for examining the topography of mutational signatures.

**Figure 6.**
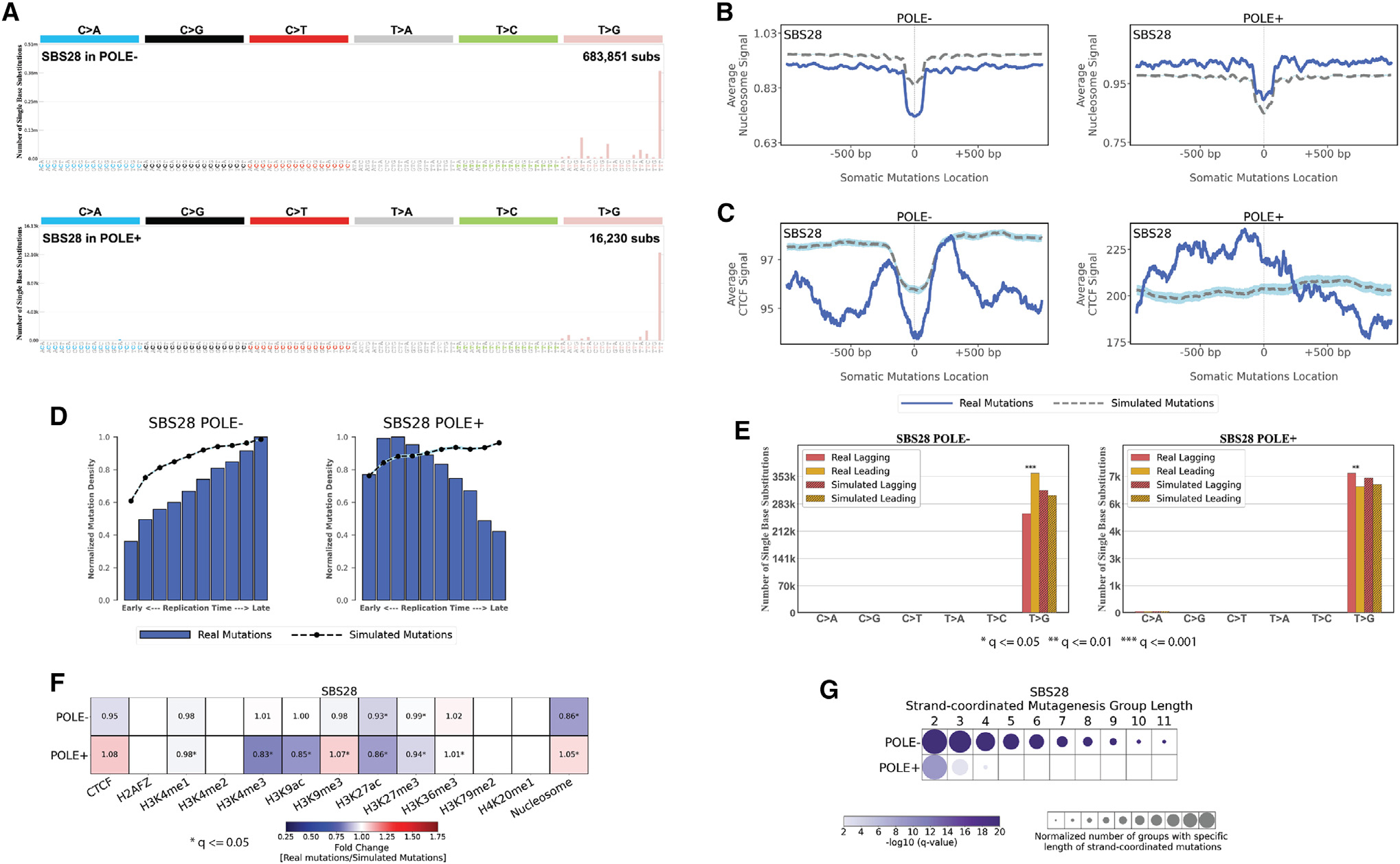
Topography of signature SBS28 in *POLE*-deficient (*POLE*^−^) and POLE-proficient (POLE^+^) samples (A) Mutational patterns of signature SBS28 in *POLE*^−^ and *POLE*^+^ samples displayed using the conventional 96 mutational classification schema for SBSs. (B) Nucleosome occupancy of SBS28 in *POLE* and *POLE*^+^ samples. Blue solid lines and gray dashed lines display the average nucleosome signal (y axes) along a 2 kb window (x axes) centered at the mutation start site for real and simulated mutations, respectively. The mutation start site is annotated in the middle of each plot and denoted as 0. The 2 kb window encompasses 1,000 base pairs 5′ adjacent to each mutation as well as 1,000 base pairs 3′ adjacent to each mutation. (C) CTCF occupancy of SBS28 in *POLE*^−^ and *POLE*^+^ samples. Blue solid lines and gray dashed lines display the average CTCF binding signal (y axes) along a 2 kb window (x axes) centered at the mutation start site for real and simulated mutations, respectively. The mutation start site is annotated in the middle of each plot and denoted as 0. The 2 kb window encompasses 1,000 base pairs 5′ adjacent to each mutation as well as 1,000 base pairs 3′ adjacent to each mutation. (D) Replication timing of SBS28 mutations in *POLE*^−^ and *POLE*^+^ samples. Replication time data are separated into deciles, with each segment containing exactly 10% of the observed replication time signal (x axes). Normalized mutation densities per decile (y axes) are presented for early (left) to late (right) replication domains. Normalized mutation densities of real somatic mutations and simulated somatic mutations from early- to late-replicating regions are shown as blue bars and dashed lines, respectively. (E) Replication strand asymmetry of SBS28 mutations in *POLE*^−^ and *POLE*^+^ samples. Bar plots display the number of mutations accumulated on the lagging strand and on the leading strand for six substitution subtypes based on the mutated pyrimidine base C>A, C>G, C>T, T>A, T>C, and T>G in red and yellow colors, respectively. Simulated mutations on lagging and leading strands are displayed in shaded bar plots. Statistically significant strand asymmetries are shown with stars: adjusted p values: *p ≤ 0.05; **p ≤ 0.01; ***p ≤ 0.001 (Fisher’s exact test corrected for multiple testing using Benjamini-Hochberg). (F) Enrichments and depletions of SBS28 somatic mutations in *POLE*^−^ and *POLE*^+^ samples within CTCF binding sites, histone modifications, and nucleosome occupied regions. Red colors correspond to enrichments of real somatic mutations when compared to simulated data. Blue colors correspond to depletions of real somatic mutations when compared to simulated data. The intensities of red and blue colors reflect the degree of enrichments or depletions based on the fold change. White colors correspond to lack of data for performing statistical comparisons. Statistically significant enrichments and depletions are annotated with an asterisk (*; adjusted p value ≤ 0.05, z-test combined with Fisher’s method and corrected for multiple testing using Benjamini-Hochberg). (G) Strand-coordinated mutagenesis of SBS28 mutations in *POLE*^−^ and *POLE*^+^ samples. Rows represent SBS28 signature in *POLE*^−^ and *POLE*^+^ samples across all cancer types and columns reflect the lengths, in numbers of consecutive mutations, of strand-coordinated mutagenesis groups. Statistically significant strand-coordinated mutagenesis (adjusted p value ≤ 0.05, z-test corrected for multiple testing using Benjamini-Hochberg) are shown as circles under the respective group length with a length starting from 2 to 11 consecutive mutations. The size of each circle reflects the number of consecutive mutation groups for the specified group length normalized for each SBS28 signature in *POLE*^−^ and *POLE*^+^ samples. The color of each circle reflects the statistical significance of the number of subsequent mutation groups for each group length with respect to the simulated mutations using −log_10_ (q value).

First, analysis of SBS28 in *POLE*-deficient samples (*POLE*^−^) and POLE proficient samples (*POLE*^+^) revealed a distinct behavior (Figure 6). While the trinucleotide patterns of SBS28 in *POLE*^+^ and *POLE*^−^ samples were similar (cosine similarity: 0.96), SBS28 in POLE^−^ samples accounted for 97.7% mutations of all SBS28 mutations, and it exhibited a clear enrichment in late-replicating regions as well as depletions at nucleosomes and at CTCF binding sites (Figures 6B–6D and 6F). Moreover, SBS28 in *POLE*^−^ samples showed a strong replication strand bias on the leading strand and exhibited a strand-coordinated mutagenesis with as many as 11 consecutively mutated substitutions (Figures 6E and 6G). In contrast, SBS28 in *POLE*^+^ samples were enriched in early replication regions, lacked depletion of mutations at nucleosomes or CTCF binding sites, had weak replication strand bias on the lagging strand, and did not exhibit much of a strand-coordinated mutagenesis (Figure 6). Based on these topographical differences, we have now split SBS28 into two distinct signatures: (1) SBS28a due to *POLE* deficiency found in ultra-hypermutate colorectal and uterine cancers and (2) SBS28b with unknown etiology found in lung and stomach cancers.

**Figure 7.**
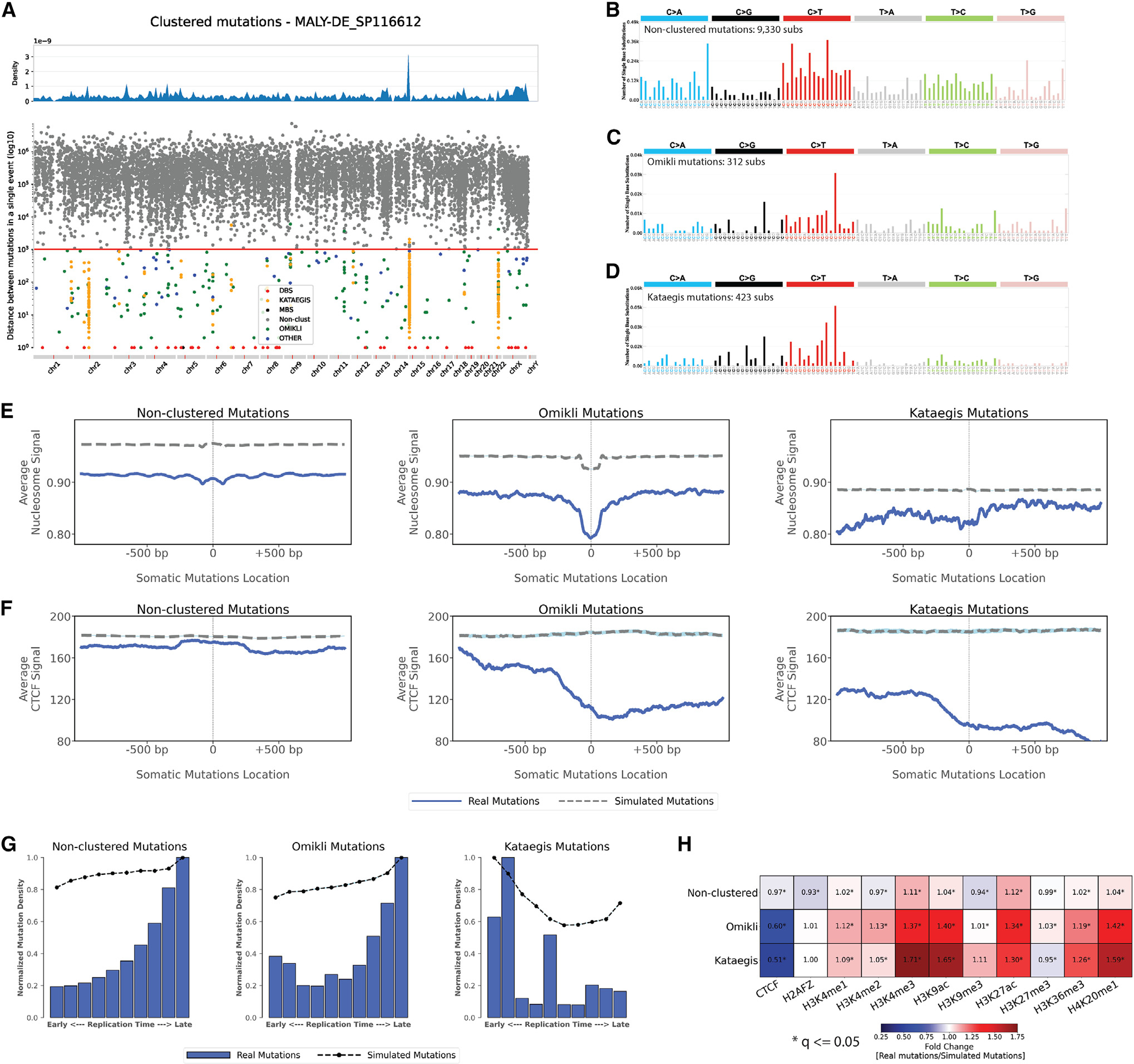
Topography of non-clustered, *omikli*, and *kataegis* substitutions across 288 whole-genome-sequenced B cell malignancies (A) A rainfall plot of an example B cell malignancy sample, MALY-DE_SP116612, depicting the intra-mutational distance (IMD) distributions of substitutions across genomic coordinates. Each dot represents the minimum distance between two adjacent mutations. Dots are colored based on their corresponding classifications. Specifically, non-clustered mutations are shown in gray, DBSs in red, multi-base substitutions (MBSs) in black, *omikli* events in green, kataegis events in orange, and all other clustered events in blue. The red line depicts the sample-dependent IMD threshold for each sample. Specific clustered mutations may be above this threshold due to corrections for regional mutation density. (B–D) The trinucleotide mutational spectra for the different catalogs of non-clustered, *omikli*, and *kataegis* mutations for the exemplar sample (DBSs and MBSs are not shown). (E) Nucleosome occupancy of non-clustered, *omikli*, and *kataegis* mutations of B cell malignancies. Blue solid lines and gray dashed lines display the average nucleosome signal (y axes) along a 2 kb window (x axes) centered at the mutation start site for real and simulated mutations, respectively. The mutation start site is annotated in the middle of each plot and denoted as 0. The 2 kb window encompasses 1,000 base pairs 5′ adjacent to each mutation as well as 1,000 base pairs 3′ adjacent to each mutation. (F) CTCF occupancy of non-clustered, *omikli*, and *kataegis* mutations of B cell malignancies. Blue solid lines and gray dashed lines display the average CTCF signal (y axes) along a 2 kb window (x axes) centered at the mutation start site for real and simulated mutations, respectively. The mutation start site is annotated in the middle of each plot and denoted as 0. The 2 kb window encompasses 1,000 base pairs 5′ adjacent to each mutation as well as 1,000 base pairs 3′ adjacent to each mutation. (G) Replication timing of non-clustered, *omikli*, and *kataegis* mutations of B cell malignancies. Replication time data are separated into deciles, with each segment containing exactly 10% of the observed replication time signal (x axes). Normalized mutation densities per decile (y axes) are presented for early (left) to late (right) replication domains. Normalized mutation densities of real somatic mutations and simulated somatic mutations from early- to late-replicating regions are shown as blue bars and dashed lines, respectively. (H) Enrichments and depletions of non-clustered, *omikli*, and *kataegis* mutations of B cell malignancies within CTCF binding sites and histone modifications. Red colors correspond to enrichments of real somatic mutations when compared to simulated data. Blue colors correspond to depletions of real somatic mutations when compared to simulated data. The intensities of red and blue colors reflect the degree of enrichments or depletions based on the fold change. White colors correspond to lack of data for performing statistical comparisons. Statistically significant enrichments and depletions are annotated with an asterisk (*; adjusted p value ≤0.05, z-test combined with Fisher’s method and corrected for multiple testing using Benjamini-Hochberg). See also Figure S5.

Second, our analyses revealed striking differences in topographical features of clustered and non-clustered somatic mutations in 288 whole-genome-sequenced B cell malignancies.^4^ In particular, the topographical behaviors of SBSs were examined after separating them into non-clustered mutations, diffuse hypermutation of substitutions termed *omikli*,^62^ and longer clusters of strand-coordinated substitutions termed *kataegis*.^34,35,63^ In contrast to most cancer types, where *omikli* and *kataegis* are predominately generated by APOBEC3 deaminases,^64^ in B cell malignancies, these clustered events are almost exclusively imprinted by the activity of AID.^64^ Further, the overall pattern of non-clustered mutations was very different than the ones of *omikli* or *kataegis*. A representative example is provided using a single malignant B cell lymphoma (Figure 7A) where non-clustered and clustered mutations have very different trinucleotide patterns (Figures 7B–7D). Non-clustered mutations exhibited different topographical features when compared to *omikli* or *kataegis*. Specifically, while non-clustered mutations had some minor periodicity in regard to nucleosome occupancy, such periodicity was not observed for any type of clustered events (Figure 7E). Similarly, non-clustered mutations were slightly depleted around CTCF binding sites, while *omikli* and *kataegis* were very highly depleted (Figures 7F and 7H). Further, non-clustered and *omikli* events were clearly enriched in late replication regions, while kataegis was highly enriched in early replication regions (Figure 7G). Distinct patterns of enrichments were also observed for both *omikli* and kataegis mutations in the vicinity of promoter and enhancer sites delineated by histone marks of H3K4me3, H3K9ac, H3K27ac, H3K36me3, and H4K20me1(Figure 7H). Only very minor differences were observed for transcription or replication strand asymmetries between clustered and non-clustered somatic mutations across the 288 whole-genome-sequenced B cell malignancies (Figure S5).

In summary, in this resource, we have performed a comprehensive topography analysis of mutational signatures encompassing 82,890,857 somatic mutations in 5,120 whole-genome-sequenced tumors integrated with 516 tissue-matched topographical features from the ENCODE project. Our evaluation encompassed examining the effects of nucleosome occupancy, histone modifications, CTCF binding sites, replication timing, transcription strand asymmetry, and replication strand asymmetry on the accumulation of somatic mutations from more than 70 distinct mutational signatures. The results from these analyses have been provided as an online resource as a part of COSMIC signatures database, https://cancer.sanger.ac.uk/signatures/, where researchers can explore each mutational signature as well as each topographical feature in a cancer-specific manner.

### Limitations of the study

As in the majority of previous examinations, the performed topography analyses relied on previously generated ENCODE experimental datasets for the presence or absence of each topographical feature. Thus, these topographical features were mapped in samples unrelated to the examined cancers and do not provide a perfect representation of the genome topography throughout the lineage of a cancer cell. Future studies will be required to evaluate whether genome topography changes during cancer evolution and whether these changes have any effect on the accumulation of mutations from different mutational processes.

## STAR★METHODS

Detailed methods are provided in the online version of this paper and include the following:

### RESOURCE AVAILABILITY

#### Lead contact

Further information and requests for resources should be directed to and will be fulfilled by the lead contact, Ludmil B. Alexandrov (L2alexandrov@health.ucsd.edu).

#### Materials availability

This study did not generate new unique reagents beyond the analyzed data and the developed source code (see below).

#### Data and code availability

- All topographical data and figures regarding topography of mutational signatures in human cancer generated in this study have been deposited at COSMIC, Catalog of Somatic Mutations in Cancer (https://cancer.sanger.ac.uk/signatures/), through COSMIC Signatures v3.3, released on May 27^th^, 2022 and are currently publicly available.
- All original Python code has been deposited on GitHub and is publicly available as of the date of publication. Links to GitHub repositories are listed in the key resources table.
- This paper analyses existing publicly available datasets. Accession numbers for the datasets are listed in the key resources table.
- Any additional information required to reanalyse the data reported in this paper is available from the lead contact upon request.

### METHOD DETAILS

#### Simulating synthetic cancer datasets

Synthetic cancer datasets were simulated using SigProfilerSimulator.^66^ Briefly, the tool randomly generated single base substitutions (SBSs), doublet base substitutions (DBSs), and small insertions and deletions (IDs) while maintaining the patterns of the original somatic mutations in each sample at a preselected resolution. Simulations were performed 100 times for each examined cancer genome while maintaining the mutational burden on each chromosome in each sample. All simulations were performed using SBS-96, DBS-78, and ID-83 mutational classification schemas.^65^ Briefly, SBS-6 represents single base substitutions in 6 mutational classes (C>A, C>G, C>T, T>A, T>C, T>G) considering the pyrimidine base of the Watson-Crick base-pair for each somatic mutation. SBS-96 is a further expansion of SBS-6 mutational classification by adding the immediate 5′ and-3′ adjacent bases for each somatic mutation within the representation of every mutation. DBS-78 catalogs doublet-base substitutions in 78 mutational channels using the maximum pyrimidine context of the Watson-Crick base-pairs,^65^ whereas ID-83 classifies small insertions and deletions into 83 mutational channels by considering the size of the ID event and the repeat size surrounding the insertion or deletion event.^65^

#### Assigning signature probabilities to somatic mutations

The performed topography analyses are based on the assignment of signature probabilities to each individual somatic mutation. For this purpose, SigProfilerExtractor was utilized for *de novo* extraction of mutational signatures and decomposition of *de novo* extracted signatures to the set of reference COSMIC mutational signatures.^4,5^ After Poisson resampling and normalization of the original mutational matrix for each replicate, SigProfilerExtractor performs nonnegative matrix factorization for multiple iterations to identify an optimal solution. Briefly, SigProfilerExtractor identifies the optional decomposition rank *k* by performing decompositions with different ranks and applying consensus clustering to identify a stable solution that best explains the underlying data.^4,5^ After extracting *de novo* mutational signatures, each *de novo* signature is matched to a COSMIC mutational signature and the COSMIC signatures are assigned using a penalized nonnegative least square approach.^4,5^ Moreover, SigProfilerExtractor automatically assigns a probability for each operative signature to generate every individual mutation within all examined samples.^4,5^

#### Matching cancer types with ENCODE datasets

Experimental data were downloaded from ENCODE for each evaluated topographical feature (Table S1). When multiple ENCODE datasets were used for the same topographical feature in a cancer type, analyses were performed for all ENCODE datasets and the results were averaged across the examined datasets. Any ENCODE genomic coordinates reported using GRCh38 annotations were first remapped to GRCh37 annotations using liftOver with exclusion of any ambiguously mapping regions.^67^ ENCODE files in bigWig file format were converted into wig files using bigWigToWig file format conversion software.^67^

We analyzed a total of 82,890,857 somatic mutations (79,269,539 single base substitutions, 429,179 doublet-base substitutions, and 3,192,139 small insertions and deletions) from 40 cancer types derived using 5,120 whole-genome sequenced samples from PCAWG, PCAWG other, and MUTOGRAPHS projects.^4,7^ Cancer types were matched to the closest available ENCODE datasets (Table S1). Topography analyses were performed both across all cancer types as well as within each individual cancer-type. The global pan-cancer analyses are shown in the manuscript while all individual cancer-type analyses are available through the COSMIC database: https://cancer.sanger.ac.uk/signatures/

#### Annotating somatic mutations based on cellular transcription

Somatic mutations were called with respect to + strand of the reference genome and annotated in regard to the pyrimidine base of the mutated base pair.^65^ Specifically, SigProfilerMatrixGenerator^65^ was used for examining transcriptional strand asymmetry for single base substitutions, doublet base substitutions, and small insertions and deletions. The tool evaluates whether a mutation occurs on the transcribed or the non-transcribed strand of well-annotated protein coding genes of the human reference genome. Mutations found in the transcribed regions of the human genome are further subclassified as *transcribed* or *un-transcribed*. Any mutations in bidirectionally transcribed regions were ignored in the current analysis. Additionally, mutation found outside the transcribed regions of the human genome are subclassified as *non-transcribed*. In all cases, mutations were oriented based on the reference strand and their pyrimidine context.

#### Annotating somatic mutations based on cellular replication

For each cancer type of interest, genomic regions were annotated either as being on the leading or being on the lagging strand using our previously developed approach.^21^ Briefly, analyses were performed for tissue-matched wavelet-smoothed replication timing signal data incorporated with valleys (replication termination zones) and peaks (replication initiation zones) data. Valleys and peaks were sorted with respect to their genomic coordinate in ascending order. Each consecutive stretches of DNA of at least 10 kilobases long with positive slope corresponded to leading strand regions on the positive strand, whereas negative slope provided lagging strand regions on the positive strand. We discarded the latest 25 kilobases of the replication termination zones to be stringent in our annotations. Having annotated genome regions as leading regions (+slope) and lagging regions (− slope) on the positive strand, we automatically acquired leading regions (− slope) and lagging regions (+slope) on the negative strand. Mutations were counted as being on leading strand or lagging strand based on their occupancy in a leading or lagging region. Similar to the annotation for transcription, in all cases, mutations were first oriented based on the reference strand and their pyrimidine context.

#### Detecting strand asymmetries across cancer types

For each mutational signature and for all cancer types having this mutational signature, we retrieved the number of mutations on each strand/region in six mutational channels (C>A, C>G, C>T, T>A, T>C, and T>G). p values were calculated for the odds ratio between the ratio of real mutations and the ratio of simulated mutations. Specifically, for transcription strand asymmetry odds ratios were calculated between the ratios of real mutations and the ratios of simulated mutations, where each ratio is calculated using the number of mutations on the transcribed strand and the number of mutations on the untranscribed strand. Similarly, for replication strand asymmetry odds ratios were calculated between the ratios of real mutations and the ratios of simulated mutations, where each ratio is calculated using the number of mutations on the lagging strand and the number of mutations on the leading strand. Lastly, for genic and intergenic regions, odds ratios were calculated between the ratios of real mutations and the ratios of simulated mutations, where each ratio is calculated using the number of mutations in the genic regions and the number of mutations in the intergenic regions. p values were computed using Fisher’s exact test and corrected for multiple testing using Benjamini-Hochberg method. Only strand asymmetries with corrected p value ≤ 0.05 and odds ratios above 1.10 were considered and reported as part of the presented results.

#### Detecting strand-coordinated mutagenesis of mutational signatures

Analyses of strand-coordinated mutagenesis searched for consecutive single base substitutions on the same DNA strand with intermutational distance less than 10,000 base-pairs within the same sample as previously done for breast cancer in.^21^ To find the consecutive mutations, all the single base substitutions in a sample were first assigned to the SBS signature with the highest probability, and only the mutations with the probability greater than or equal to a pre-set cut-off value of at least 0.50 were retained (*i.e*., at least 50% chance for a signature to have generated that mutation). Somatic mutations were sorted in an ascending order in regard to chromosomal positions and consecutive groups of substitutions with the same mutational context, on the same DNA strand, and attributed to the same substitution signature were identified. Where applicable, consecutive groups of substitutions were combined with the appropriate adjustments of their group lengths. Any consecutive groups of substitutions with length of 1 were discarded. All results coming across different samples were pooled. For each SBS signature and strand-coordinated mutagenesis group length, the observed number of groups for real mutations and the expected number of groups coming from 100 simulated datasets were compared with the simulated datasets serving as null hypotheses. p values were computed using z-tests evaluating whether the mean values of the expected number of groups for each simulated dataset were equal to the mean values of the observed number of groups for each observed dataset. The computed p values were corrected for multiple testing using Benjamini-Hochberg method. SBS signatures and strand-coordinated mutagenesis group lengths with corrected p value ≤ 0.05 were considered and reported as part of the presented results.

#### Analyses of replication timing

As previously done in breast cancer, wavelet-smoothed signal data were used in the replication timing analysis.^21^ Briefly, cancer types were matched with ENCODE data from the most suitable tissue or cell line and the corresponding Repli-seq dataset was utilized in the analysis (Table S1). Given the Repli-Seq signal data for a tissue of interest, a higher replication time signal reflects an earlier replication timing. The replication time signals were each sorted in a descending order and, subsequently, the sorted replication time signals were divided into deciles. Each decile contains approximately 10% of each replication time signal. Somatic mutations of interest were distributed within the corresponding deciles based on their overlap with the replication domains in the examined deciles. To correct for genomic size, mutation densities were calculated by dividing the numbers of somatic mutations within each decile by the number of attributable bases of adenine, thymine, guanine, and cytosine (excluding any ambiguous genomic annotations in the reference genome). To compare replication timing of different mutational signatures with each other, mutation densities were further normalized with respect to the highest mutation density observed for each respective signature. Lastly, as with other analyses, the reported replication timing analyses included only signatures with at least 1,000 somatic mutations unambiguously attributed to an individual mutational signature.

#### Mutational signature and cancer type specific replication timing analysis

To compare the replication timing between real and simulated somatic mutations, cancer-type specific normalized mutation densities were calculated for real mutations, $x_{\text{real}}={\left[x_{\text{real}}^{1},x_{\text{real}}^{2},\ldots ,x_{\text{real}}^{10}\right]}^{⊤}$, and for each of the 100 simulated synthetic cancer datasets, $X_{\text{sim\_i}}={\left[x_{\text{sim\_i}}^{1},x_{\text{sim\_i}}^{2},\ldots ,X_{sim\_i}^{10}\right]}^{⊤}$, where ${sim}_{-}i=1,2,\ldots ,100$. Normalized mutation density vectors generated based on each simulated dataset were combined and the matrix $X_{\text{sims}}={\left[X_{\text{sim\_1}},X_{\text{sim\_2}},\ldots ,X_{\text{sim\_100}}\right]}^{⊤}$ was generated. Mean simulated vector, ${\overset{‾}{X}}_{\text{sims}}$, standard deviation vector, $\sigma_{\text{sims}}^{x}$, and their 95% confidence intervals were calculated using SciPy. As a result, normalized mutation densities of real mutations across replication timing deciles, $x_{\text{real}}$; were compared to the averaged normalized mutation densities of simulated mutations across replication timing deciles, ${\overset{‾}{x}}_{\text{sims}}$. Results were further averaged across all cancer types in order to generate the summary plots presented in the manuscript.

To classify whether the mutation density was increasing, flat, or decreasing in regards to replication timing, we fitted a linear regression model to the values of the normalized mutation densities, $x_{\text{real}}$. A mutational signature was considered to be increasing from early to late replicating regions if the slope m was statistically significant from a flat line and the values of $x_{\text{real}}$ were monotonically increasing. A mutational signature was considered to be decreasing from early to late replicating regions if the slope m was statistically significant from a flat line and the values of $x_{\text{real}}$ were monotonically decreasing. Lastly, a mutational signature was considered to be generally unaffected by replication timing if the slope m was not statistically significant from a flat line.

#### Occupancy analysis of topographical features

Genomic occupancy analysis evaluated the relationship between mutational signatures and the genomic locations of different topographical features, including: *(i)* nucleosomes; *(ii)* transcription factors; and *(iii)* histone modifications. Specifically, occupancy analysis of topographical features focused on mutations within a specific genomic window, and it evaluated the average experimental signal of a particular topographical feature. In all cases, this window was centered on a somatic mutation, and the window began 1,000 base-pair (bp) 5′ of a mutation and ended 1,000 bp 3′ of a mutation; for example, this resulted in a region 2,001 bp for each examined somatic single base substitution. For a given topographical feature, the analysis evaluated the experimental signal for a set of cancer-matched datasets from ENCODE by averaging the signal across the regions of interest. For example, to evaluate the connection between SBS2 and CTCF binding in breast adenocarcinoma, our analysis utilized 4 datasets of chromatin immunoprecipitation followed by sequencing (ChIP–seq) from breast tissues in ENCODE (Table S1); for each mutation unequivocally attributed to SBS2 in the examined breast cancers, the CTCF signals within 2,001 bp around the mutation were averaged across the 4 examined datasets. As in other analysis, the reported occupancy results included only signatures with at least 1,000 unequivocally attributed somatic mutations to that specific mutational signature within each examined cancer type.

Next, we averaged both the real and simulated mutations in two rounds where the first round of accumulation and averaging was across all mutations for each cancer-type matched ENCODE dataset and the second round of accumulation and averaging was across all cancer-type matched ENCODE datasets. For real mutations, in the first round, for each cancer-type matched ENCODE dataset, we accumulated the average signal vectors coming from all real somatic mutations. This resulted in a cancer-type specific vector $K_{\text{real}}={\left[k_{\text{real}}^{0},k_{\text{real}}^{1},\ldots ,k_{\text{real}}^{2000}\right]}^{⊤}$, where $K_{\text{real}}$ is the average signal of the topographical feature of interest in a 2,001 bp window using all real mutations. In the second round, we accumulated the average signal vectors $K_{\text{real}}$ that were attained in the first round coming from each cancer-type matching ENCODE dataset and derived their average for the total number of considered ENCODE datasets. This results in a global vector $M_{\text{real}}={\left[m_{\text{real}}^{0},{\;m}_{\text{real}}^{1},\ldots ,m_{\text{real}}^{2000}\right]}^{⊤}$, where $M_{\text{real}}$ represents the average signal of the topographical feature in a 2,001 bp window using all cancer-type matching ENCODE datasets. The same procedure was repeated for each of the 100 simulated synthetic cancer datasets resulting in an average $K_{\text{sims}}$ for a signature and topographical feature within each cancer type as well as an average $M_{\text{sims}}$ for a signature and topographical feature across all cancer types. For each signature and topographical feature, comparisons between $K_{\text{real}}$ and $K_{\text{sims}}$ were performed within each cancer type while a global comparison was performed between $M_{\text{real}}$ and $M_{\text{sims}}$. Linear correlations were performed to evaluate whether the occupancy signal within ±500 base-pair windows around a somatic mutation for a cancer type, $K_{\text{real}}$, is correlated with the average signal across all cancer types $M_{\text{real}}$. Any statistically significant Pearson’s correlations, based on Benjamini-Hochberg corrected and z-test computed p value ≤ 0.05, were reported as part of the presented results.

#### Abundance analysis of mutational signatures and topographical features

In addition to performing occupancy analysis, our abundance analysis evaluated the enrichment, depletion, or no relationship between mutational signatures and topography features of interest. Specifically, we investigated the relationships between mutational signatures and the following topographical features: *(i)* nucleosomes*; (ii)* transcription factors; and *(iii)* histone modifications. For each mutational signature and topography feature, we examined whether there is an enrichment, depletion, or no statistically significant relation between the mutational signature and the topography feature of interest by comparing the real somatic mutations with the sets of simulated somatic mutations. Specifically, the average signal vector of real mutations for each cancer-type matched ENCODE dataset, $K_{\text{real}}$, was obtained as described in the occupancy analysis. An average value, $S_{\text{real}}$; was derived for ±50 bp window centered at the somatic mutations for each cancer-type matched ENCODE dataset. Similar analysis was performed for the 100 simulated cancer datasets, which allowed deriving a $K_{\text{sim}}$ average vector and $s_{\text{sim}}$ average value.

To evaluate whether this average signal value of real mutations, $s_{\text{real}}$, was expected by chance given the average signal values coming from 100 simulations, $s_{\text{sim\_i}}$ for i=1,2,…,100, we assessed the statistical significance of each fold change and associated it with a p value. Average signal value for real mutations was determined as observed value, $S_{\text{real}}$, and average signal values coming from 100 simulations were determined as expected values, ${\left[S_{\text{sim\_1}},S_{\text{sim\_2}},\ldots ,S_{\text{sim\_1}}100\right]}^{⊤}$. Z-test was applied to test whether the observed value was the mean of expected values under null hypothesis and a p value together with a test statistic were obtained. The computed p values were corrected for multiple testing using Benjamini-Hochberg method and only p values ≤ 0.05 were considered and reported as part of the presented results. In case of multiple ENCODE datasets availability for a certain cancer type and topography feature of interest, the calculated p values coming from each cancer-type matched ENCODE dataset were pooled and combined using Fisher’s method. In this cases, p values were corrected for multiple testing using Benjamini-Hochberg method after combining. Likewise, calculated fold changes acquired from each ENCODE dataset were averaged and average fold change was obtained.

#### Classifying cancers into ones with low and ones with high APOBEC3 mutations

Each cancer sample with operative APOBEC3-associated signatures SBS2 or SBS13 was classified as either having low, mid, or high APOBEC3 presence. Specifically, we utilized a previously developed scheme for calculating APOBEC3 presence,^73^ where a ratio was derived as the natural logarithm of the total number of mutations attributed to the APOBEC3-associated signatures SBS2 or SBS13 divided by the natural logarithm of the total number of mutations excluding ones due to APOBEC3-associated signatures SBS2 or SBS13. Thus, for each sample the ratio was calculated in the following manner:

$$ratio=\frac{{log}_{e}(\text{number of mutations attributed to SBS2 or SBS13})}{{log}_{e}(\text{total number of mutations NOT attributed to SBS2 and SBS13})}$$

Cancers with ratios ≥ 0.90 were classified as samples with high APOBEC3 presence and cancers with ratios ≥ 0.75 were classified as samples with low APOBEC3 presence. All samples with ratios between 0.75 and 0.90 were classified as mid APOBEC3 presence and were not considered in our subsequent replication timing re-analysis. Specifically, we repeated the replication timing for all cancer types by separately examining samples with low APOBEC3 presence and separately examining samples with high APOBEC3 presence. As done in the prior analysis in this manuscript, all results were averaged within and across the examined cancer types.

### QUANTIFICATION AND STATISTICAL ANALYSIS

All statistical analysis were performed in Python using NumPy, SciPy, statsmodels, and other standard statistical modules. Statistical significance was analyzed by Fisher’s exact test and z-test. Where applicable, p values were combined with Fisher’s method. All p values were corrected for multiple testing using Benjamini and Hochberg method. All adjusted p values of * ≤ 0.05; ** ≤ 0.01; *** ≤ 0.001 were considered significant. Statistical parameters and details of the analyses can be found in the figure legends and “method details”.

### ADDITIONAL RESOURCES

All topographical data and figures regarding topography of mutational signatures in human cancer generated in this study were deposited at COSMIC, Catalog of Somatic Mutations in Cancer (https://cancer.sanger.ac.uk/signatures/), through COSMICv3.3, May 27^th^, 2022.

## Supplementary Material

1

2

## Figures and Tables

**KEY RESOURCES TABLE:** 

REAGENT or RESOURCE	SOURCE	IDENTIFIER

Biological samples		

PCAWG project (core WGS dataset) Somatic Mutations and Mutational Catalogs from PCAWG Project	Alexandrov et al.^4^	https://www.synapse.org/#!Synapse:syn11804058 https://www.synapse.org/#!Synapse:syn11804040
PCAWG project (additional WGS dataset) VCF like sample files and SBS signatures in samples	Alexandrov et al.^4^	https://www.synapse.org/#!Synapse:syn11801872 https://www.synapse.org/#!Synapse:syn11801496
MUTOGRAPHS project	Moody et al.^7^	https://doi.org/10.6084/m9.figshare.22744733

Deposited data		

Topography of Mutational Signatures in Human Cancer	This paper	COSMIC Signatures v3.3https://cancer.sanger.ac.uk/signatures

Software and algorithms		

SigProfilerMatrixGenerator (v1.1.31)	Bergstrom et al.^65^	https://github.com/AlexandrovLab/SigProfilerMatrixGenerator
SigProfilerSimulator (v1.1.2)	Bergstrom et al.^66^	https://github.com/AlexandrovLab/SigProfilerSimulator
SigProfilerExtractor (v1.1.0)	Islam et al.^5^	https://github.com/AlexandrovLab/SigProfilerExtractor
SigProfilerClusters (v1.0.11)	Bergstrom et al.^63^	https://github.com/AlexandrovLab/SigProfilerClusters
SigProfilerTopography (v1.0.70)	This paper	https://github.com/AlexandrovLab/SigProfilerTopography
Cancer-type specific and across all cancer-types combined topography analysis	This paper	https://github.com/AlexandrovLab/SigProfilerTopographyCombined
bigWigToWig tool (v446 April 2023)	Kent et al.^67^	http://hgdownload.cse.ucsc.edu/admin/exe/
liftOver tool (v446 April 2023)	Kent et al.^67^	http://hgdownload.cse.ucsc.edu/admin/exe/
Python (v3.7.0)	Python Software Foundation	https://www.python.org/
Python package: pandas (v1.1.5)	McKinney^68^	https://pandas.pydata.org
Python package: NumPy (v1.20.1)	Harris et al.^69^	https://numpy.org
Python package: matplotlib (v3.4.2)	Hunter^70^	https://matplotlib.org
Python package: SciPy (v1.6.3)	Virtanen et al.^71^	https://scipy.org
Python package: statsmodels (v0.12.2)	Seabold and Perktold^72^	https://www.statsmodels.org

Other		

Transcription factors (TF) binding sites datasets (TF ChIP-seq assays)	ENCODE Project	https://www.encodeproject.org/ Exact file name(s) for each utilized dataset is available as part of Table S1
Histone modifications sites datasets (Histone ChIP-seq assays)	ENCODE Project	https://www.encodeproject.org/ Exact file name(s) for each utilized dataset is available as part of Table S1
Nucleosome occupancy datasets (MNase-seq assays)	ENCODE Project	https://www.encodeproject.org/ Exact file name(s) for each utilized dataset is available as part of Table S1
Replication timing datasets (Repli-seq assays)	ENCODE Project	https://www.encodeproject.org/ Exact file name(s) for each utilized dataset is available as part of Table S1

## Highlights

- Mutations imprinted by mutational signatures are affected by topographical genomic features
- Mutational signatures with related etiologies are similarly affected by genomic topography
- Periodicity and cancer-type-specific enrichments/depletions are observed for some signatures
- Updated COSMIC database links 76 signatures in 40 cancer types with 516 topography features
