# Supplementary material for: Topography of mutational signatures in human cancer
Source: Cell Rep. Author manuscript; Available in PMC 2023 Sep 19. (PMC10507738; doi:10.1016/j.celrep.2023.112930)
Supplement: 1 [file NIHMS1928262-supplement-1.pdf]

Cell Reports, Volume *42*

Supplemental information

Topography of mutational signatures in human cancer

Burçak Otlu, Marcos Díaz-Gay, Ian Vermes, Erik N. Bergstrom, Maria Zhivagui, Mark Barnes, and Ludmil B. Alexandrov

Supplemental Information

Figure S1. Somatic mutations in genic and intergenic regions imprinted by different mutational signatures. Related to Figure 1.

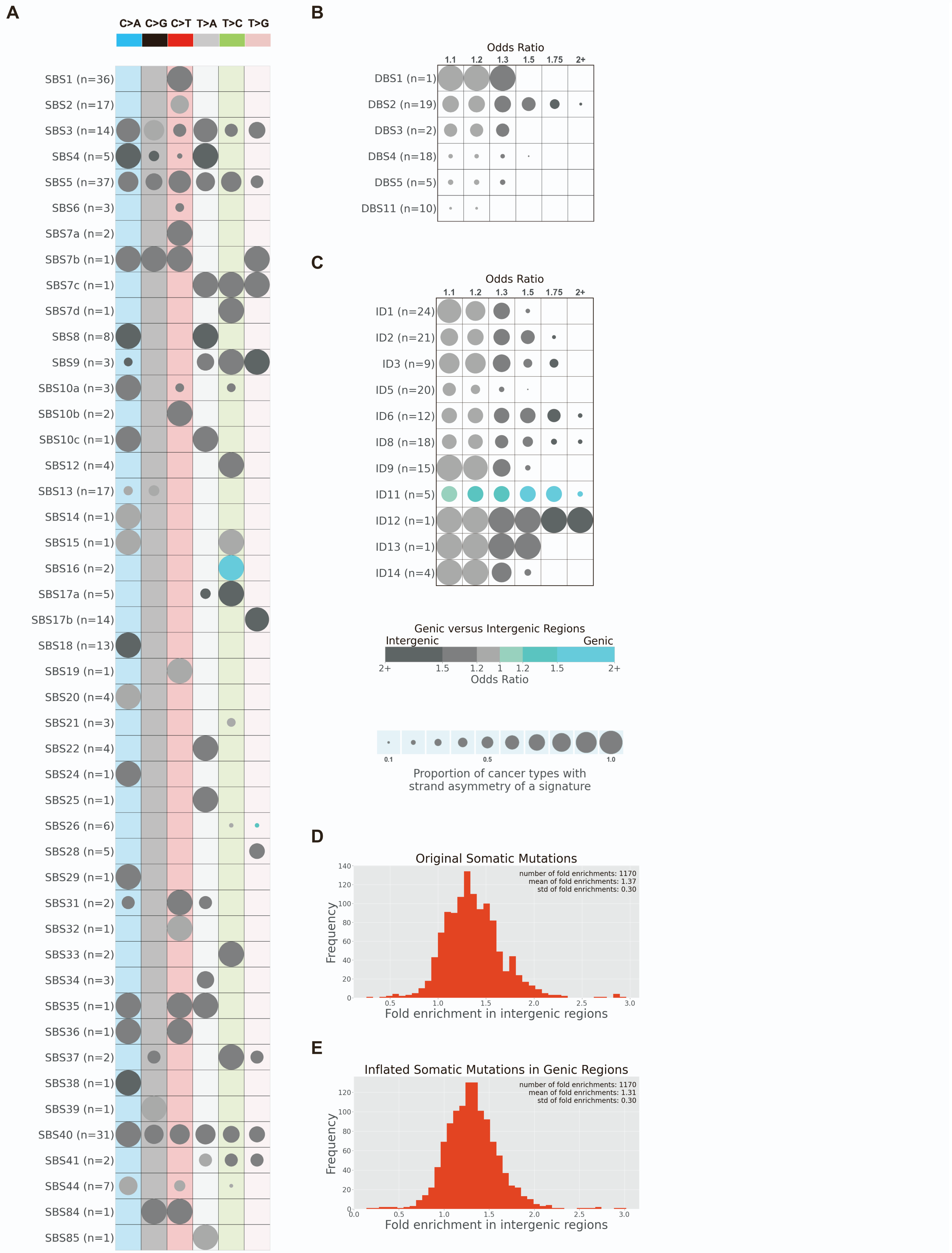

Figure S1. Somatic mutations in genic and intergenic regions imprinted by different mutational signatures. Related to Figure 1. (A) Somatic mutations in genic and intergenic regions for signatures of single base substitutions (SBSs). Rows represent the signatures, where n reflects the number of cancer types in which each signature was found. Columns display the six substitution subtypes based on the mutated pyrimidine base: C>A, C>G, C>T, T>A, T>C, and T>G. SBS signatures with genic and intergenic regions asymmetries with adjusted p-values ≤ 0.05 (Fisher's exact test corrected for multiple testing using Benjamini-Hochberg) are shown in circles with cyan and grey colours, respectively. The colour intensity reflects the odds ratio between the ratio of real mutations and the ratio of simulated mutations, where each ratio is calculated using the number of mutations in the genic regions and the number of mutations in the intergenic regions. Only odds ratios above 1.10 are shown. Circle sizes reflect the proportion of cancer types exhibiting a signature with specific genic versus intergenic regions asymmetry. **(B)** Somatic mutations in genic and intergenic regions for signatures of doublet-base substitutions (DBSs). Data are presented in a format similar to the one in panel (A). **(C)** Somatic mutations in genic and intergenic regions for small insertions/deletions (IDs). Data are presented in a format similar to the one in panel (A). **(D)** Histogram of fold enrichment as odds ratio between the ratio of real mutations and the ratio of simulated mutations, where each ratio is calculated using the number of mutations in the genic regions and the number of mutations in the intergenic regions. Frequency of fold enrichments (y-axis) are presented for discreet bins of fold enrichments (x-axis). Each fold enrichment reflects the odds ratio between real and simulated mutations where each ratio is the number of mutations in intergenic regions divided by the number of mutations in genic regions. Total number of fold enrichments, mean, and standard deviation of fold enrichments are shown in the upper right corner of the histogram. **(E)** Same format as panel (D) with the underlying data reflecting fold enrichments after inflating the number of somatic mutations in genic regions to remove any transcription strand asymmetry.

Figure S2. The effect of replication timing on mutational signatures. Related to Figure 2.

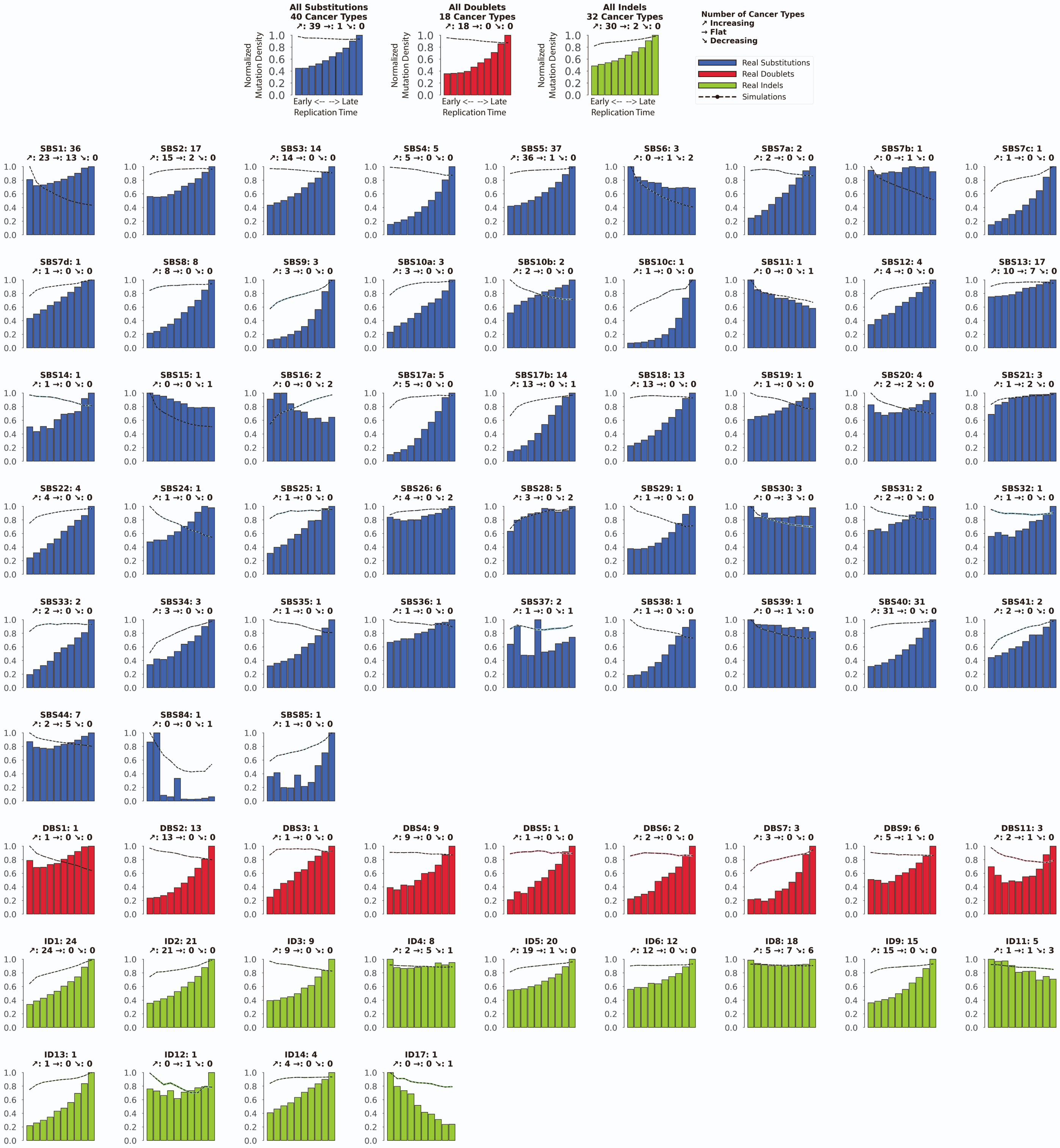

Figure S2. The effect of replication timing on mutational signatures. Related to Figure 2. Top three panels reflect results for all single base substitutions (SBSs), all doublet-base substitutions (DBSs), and all small insertions/deletions (IDs) across all examined cancer types with each cancer type examined separately. Bottom panels reflect all somatic mutations attributed to a particular signature across all cancer types. Replication time data are separated into deciles, with each segment containing exactly 10% of the observed replication time signal (x-axes). Normalized mutation densities per decile (y-axes) are presented for early (left) to late (right) replication domains. Real data for SBS signatures are shown as blue bars, for DBS signatures as red bars, and for ID signatures as green bars. In all cases, simulated somatic mutations are shown as dashed lines. The total number of evaluated cancer types for a particular mutational signature is shown on top of each plot (*e.g.,* 36 cancer types were evaluated for SBS1). For each signature, the number of cancer types where the mutation density increases with replication timing is shown next to ➚ (*e.g.,* 23 cancer types for SBS1). Similarly, the number of cancer types where the mutation density decreases with replication timing is shown next to ➘ (*e.g.,* 0 cancer types for SBS1). Lastly, the number of cancer types where the mutation density is not affected by replication timing is shown next to ➞ (*e.g.,* 13 cancer types for SBS1).

Figure S3. The effect of nucleosome occupancy on mutational signatures. Related to Figure 3.

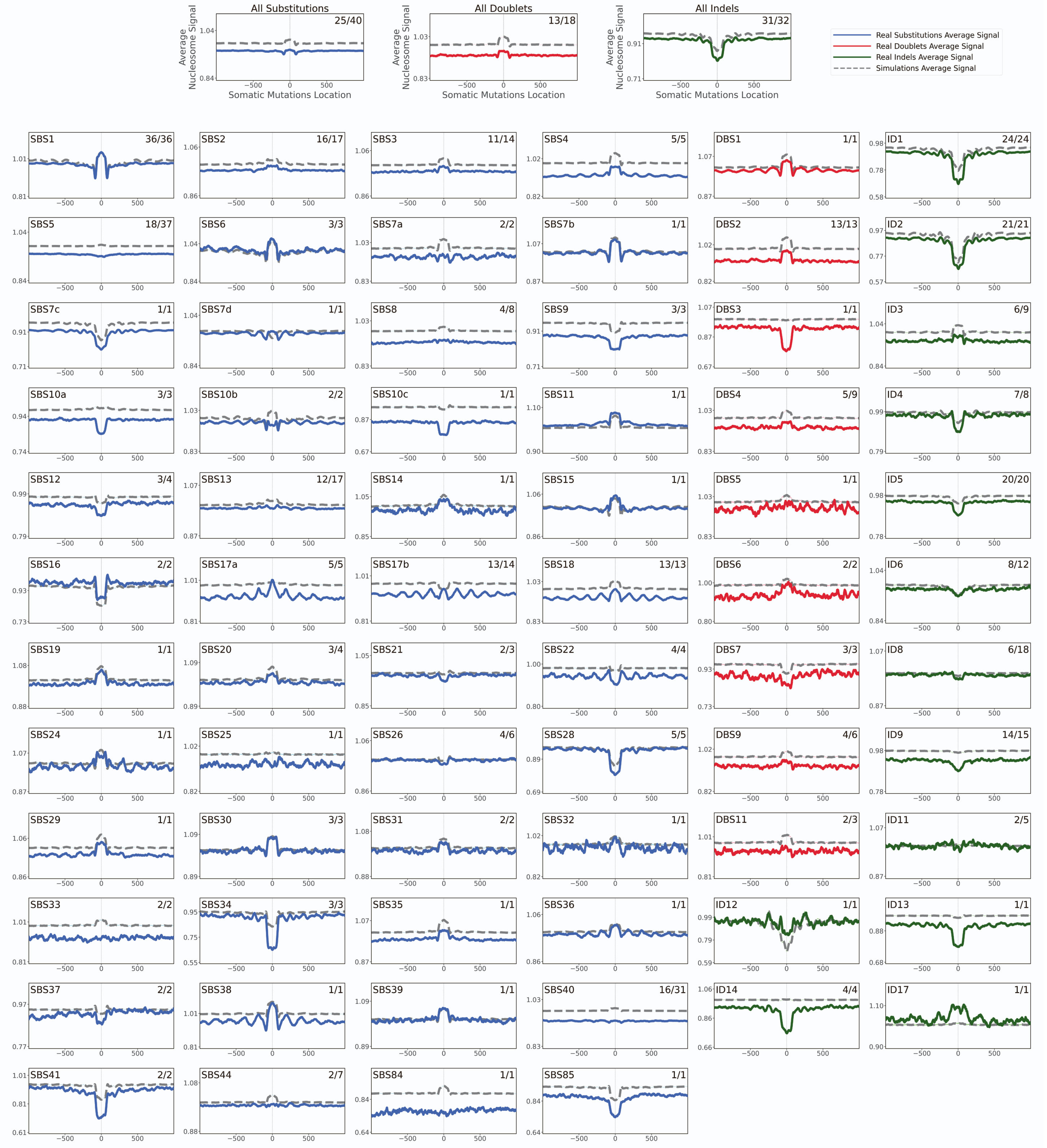

Figure S3. The effect of nucleosome occupancy on mutational signatures. Related to Figure 3. Top three panels reflect results for all single base substitutions (SBSs), all doublet-base substitutions (DBSs), and all small insertions/deletions (IDs) across all examined cancer types with each cancer type examined separately. Bottom panels reflect all somatic mutations attributed to a particular signature across all cancer types. In all cases, solid lines correspond to real somatic mutations with blue solid lines reflecting SBSs, red solid lines reflecting DBSs, and green solid lines reflecting IDs. Solid lines and dashed lines display the average nucleosome signal (y-axes) along a 2 kilobase window (x-axes) centred at the mutation start site for real and simulated mutations, respectively. The mutation start site is annotated in the middle of each plot and denoted as 0. The 2 kilobase window encompasses 1,000 base-pairs 5' adjacent to each mutation as well as 1,000 base-pairs 3' adjacent to each mutation. For each mutational signatures, the total number of similar and considered cancer types using an *X/Y* format, with *X* being the number of cancer types where a signature has similar nucleosome behaviour (Pearson correlation ≥ 0.5 and adjusted p-value ≤ 0.05, z-test corrected for multiple testing using Benjamini-Hochberg) and *Y* representing the total number of examined cancer types for that signature. For example, signature SBS3 annotated with 11/14 reflects a total of 14 examined cancer types with similar nucleosome behaviour observed in 11 of these 14 cancer types.

Figure S4. Relationships between mutational signatures and histone modifications. Related to Figure 5.

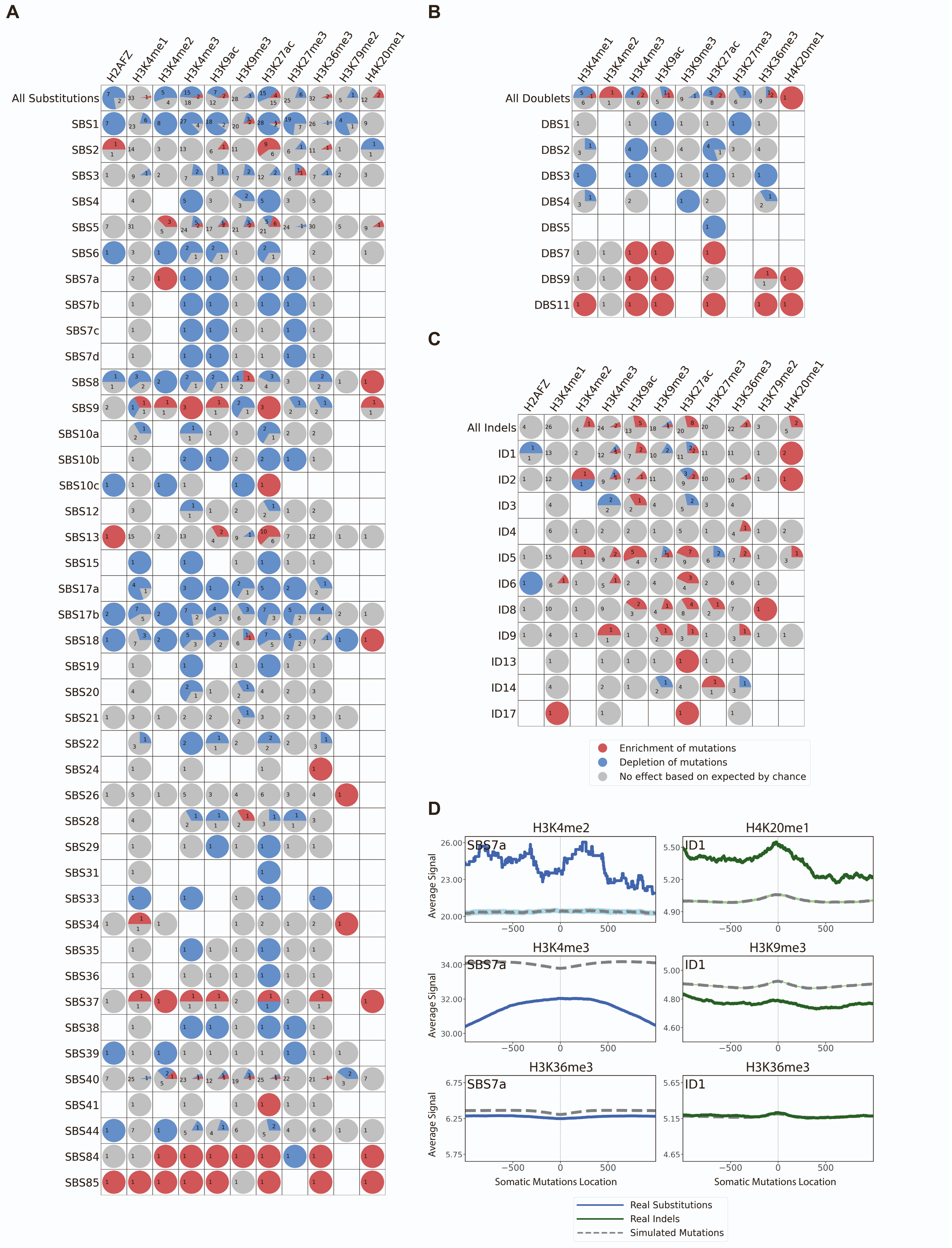

Figure S4. Relationships between mutational signatures and histone modifications. Related to Figure 5. (A-C) Relationships between 11 histone modifications and signatures of single base substitutions (SBSs) in panel (A), doublet-base substitutions (DBSs) in panel (B), and small insertions/deletions (IDs) in panel (C). The examined histone modifications encompass H2AFZ, H3K4me1, H3K4me2, H3K4me3, H3K9ac, H3K9me3, H3K27ac, H3K27me3, H3K36me3, H3K79me2, and H4K20me1. Rows and columns reflect the mutational signatures and histone modifications, respectively. The circle in each cell is separated in red, blue, and grey segments proportionate to the cancer types in which the signature has a specific behaviour. A red segment in a circle reflects the signature being enriched in the vicinity of a histone modification (adjusted p-value ≤ 0.05, z-test combined with Fisher's method and corrected for multiple testing using Benjamini-Hochberg and at least 5% enrichment). A blue segment in a circle reflects the signature being depleted in the vicinity of a histone modification (adjusted p-value ≤ 0.05, z-test combined with Fisher's method and corrected for multiple testing using Benjamini-Hochberg and at least 5% depletion). A grey segment in a circle corresponds to neither depletion nor enrichment of the signature in the vicinity of a histone modification. Cells without a circle correspond to insufficient data to perform any statistical comparisons. **(D)** Exemplars of enrichment, depletions, or no effect for several histone modifications and signatures SBS7a and ID1. Solid lines and dashed lines display the average signal for a particular histone modification (y-axes) along a 2 kilobase window (x-axes) centred at the mutation start site for real and simulated mutations, respectively. The mutation start site is annotated in the middle of each plot and denoted as 0. The 2 kilobase window encompasses 1,000 base-pairs 5' adjacent to each mutation as well as 1,000 base-pairs 3' adjacent to each mutation.

Figure S5. Strand asymmetries of non-clustered, omikli, and kataegis substitutions across 288 whole-genome sequenced B-cell malignancies. Related to Figure 7.

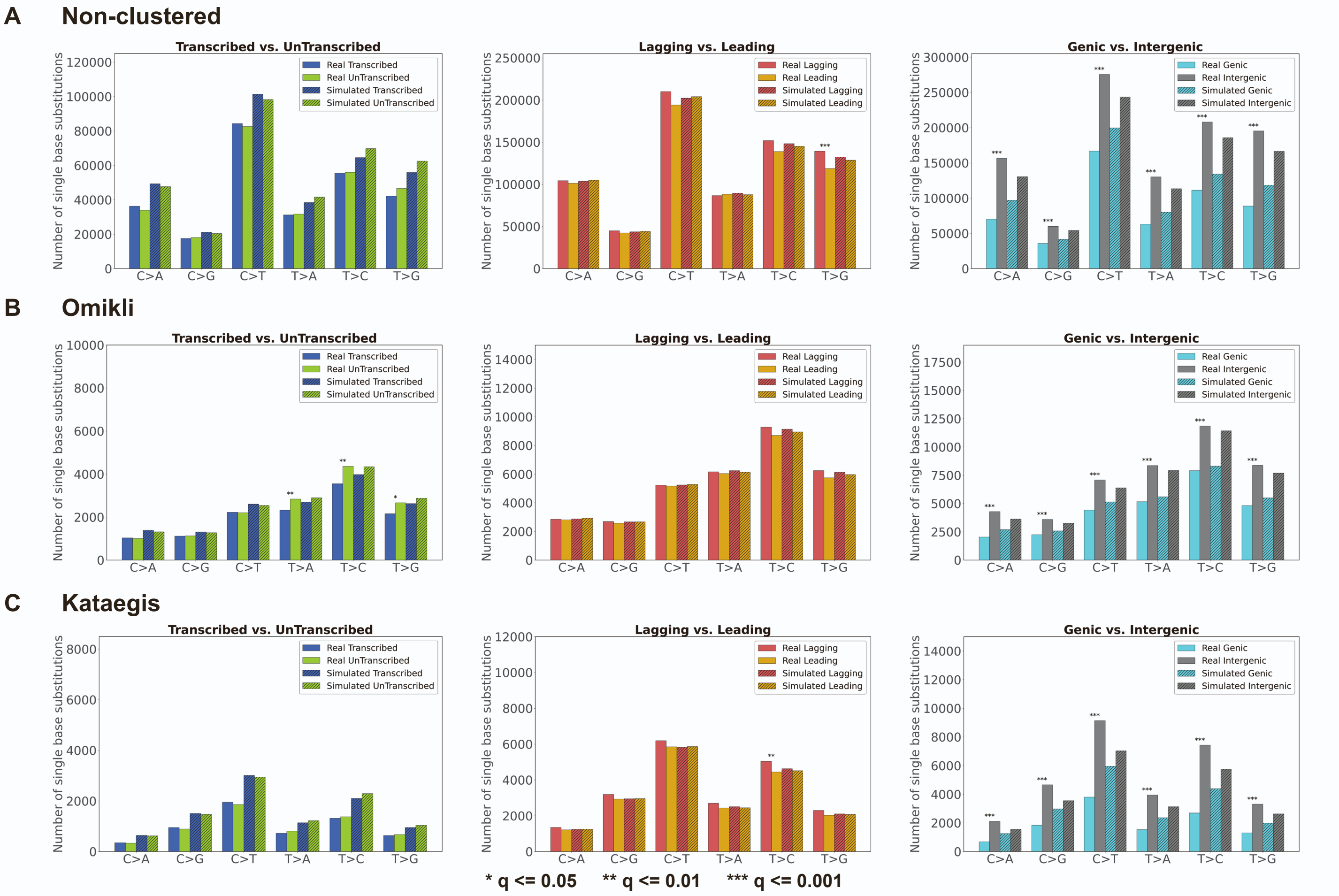

Figure S5. Strand asymmetries of non-clustered, omikli, and kataegis substitutions across 288 whole-genome sequenced B-cell malignancies. Related to Figure 7. Transcription strand asymmetries are shown in the left panels where bars display the six substitution subtypes based on the mutated pyrimidine base: C>A, C>G, C>T, T>A, T>C, and T>G (depicted on the x-axes). Y-axes correspond to the numbers of single base substitutions. Blue bars reflect real transcribed substitutions, while shaded blue bars correspond to simulated transcribed substitutions. Similarly, green bars reflect real untranscribed mutations, whereas shaded green bars correspond to simulated untranscribed substitutions. Replication strand asymmetries are shown in the middle panels where bars display the six substitution subtypes based on the mutated pyrimidine base: C>A, C>G, C>T, T>A, T>C, and T>G (depicted on the x-axes). Y-axes correspond to the numbers of single base substitutions. Red bars reflect real substitutions on the lagging strand, while shaded red bars correspond to simulated substitutions on the lagging strand. Similarly, yellow bars reflect real substitutions on the leading strand, whereas shaded yellow bars correspond to simulated substitutions on the leading strand. Comparisons of genic and intergenic regions are shown in the right panels where bars display the six substitution subtypes based on the mutated pyrimidine base: C>A, C>G, C>T, T>A, T>C, and T>G (depicted on the x-axes). Y-axes correspond to the numbers of single base substitutions. Cyan bars reflect real substitutions in genic regions, while shaded cyan bars correspond to simulated substitutions in genic regions. Similarly, grey bars reflect real substitutions in intergenic regions, whereas shaded grey bars correspond to simulated substitutions in intergenic regions. Results for non-clustered mutations are shown in panel **(A)**, omikli mutations in panel **(B)**, and kataegis mutations in panel **(C)**. Statistically significant strand asymmetries are shown with stars: adjusted p-value of * ≤ 0.05; ** ≤ 0.01; *** ≤ 0.001 (Fisher's exact test corrected for multiple testing using Benjamini-Hochberg).
